# Perceptual discrimination difficulty and familiarity in the Uncanny Valley: more like a “Happy Valley”

**DOI:** 10.3389/fpsyg.2014.01219

**Published:** 2014-11-19

**Authors:** Marcus Cheetham, Pascal Suter, Lutz Jancke

**Affiliations:** ^1^Department of Neuropsychology, University of ZurichZurich, Switzerland; ^2^Department of Psychology, Nungin UniversitySeoul, South Korea

**Keywords:** perceptual discrimination, categorical perception, categorization, uncanny valley, human likeness, other-race effect, processing fluency, mere exposure

## Abstract

The *Uncanny Valley Hypothesis* (*UVH*) predicts that greater difficulty perceptually discriminating between categorically ambiguous human and humanlike characters (e.g., highly realistic robot) evokes negatively valenced (i.e., uncanny) affect. An *ABX perceptual discrimination task* and signal detection analysis was used to examine the profile of *perceptual discrimination* (*PD*) difficulty along the UVH' *dimension of human likeness* (*DHL*). This was represented using avatar-to-human morph continua. Rejecting the implicitly assumed profile of PD difficulty underlying the UVH' prediction, Experiment 1 showed that PD difficulty was reduced for categorically ambiguous faces but, notably, enhanced for human faces. Rejecting the UVH' predicted relationship between PD difficulty and negative affect (assessed in terms of the UVH' *familiarity* dimension), Experiment 2 demonstrated that greater PD difficulty correlates with more positively valenced affect. Critically, this effect was strongest for the ambiguous faces, suggesting a correlative relationship between PD difficulty and feelings of familiarity more consistent with the metaphor *happy valley*. This relationship is also consistent with a *fluency amplification* instead of the hitherto proposed *hedonic fluency* account of affect along the DHL. Experiment 3 found no evidence that the asymmetry in the profile of PD along the DHL is attributable to a *differential processing bias* (cf. *other-race effect*), i.e., processing avatars at a category level but human faces at an individual level. In conclusion, the present data for static faces show clear effects that, however, strongly challenge the UVH' implicitly assumed profile of PD difficulty along the DHL and the predicted relationship between this and feelings of familiarity.

## Introduction

**Figure 1:**
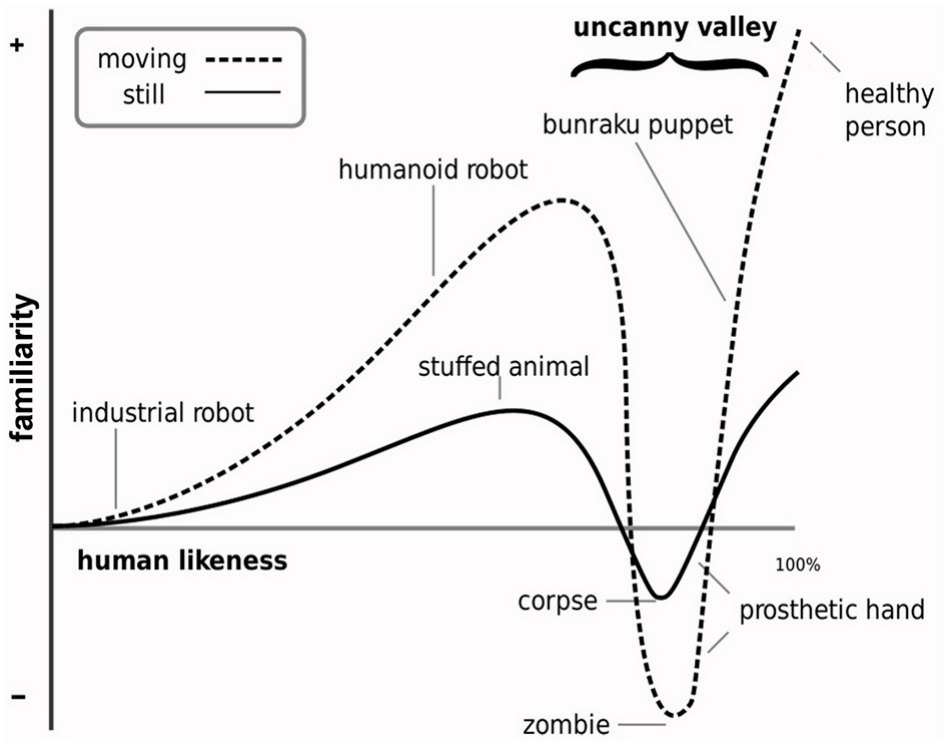
**Illustration of the Uncanny Valley Hypothesis**. The uncanny valley hypothesis proposes a non-linear relationship between affective experience and physical humanlike realism in appearance and motion (the non-linearity is more pronounced for motion). The key prediction of the hypothesis is that a high degree of human likeness will evoke a sharp negative peak (valley) in affective experience (i.e., along the *familiarity dimension*). This valley is characterized by feelings of strangeness (and the uncanny). The valley occurs at the point along the *dimension of human likeness* at which objects are categorically most ambiguous (illustration adapted from MacDorman, 2005).

Progress in robotics and computer graphics in simulating human appearance and behavior to high degrees of realism has fuelled research interest in the *Uncanny Valley Hypothesis* (*UVH*) (Mori, 1970). The UVH predicts that perceptual difficulty discriminating between highly realistic humanlike objects and characters (e.g., robot, prosthetic hand) and their human equivalent will evoke an unpleasant affective state. This state is described as one of feelings of personal disquiet, strangeness and the uncanny. These feelings are conjectured to occur at the point of realism along the UVH' *dimension of human likeness* (*DHL*) at which the attribution of objects and characters to the human or nonhuman category is subject to greatest ambiguity (i.e., the “valley” in Figure 1). Studies to date have not provided a consistent picture in favor of this uncanny effect, but this field of research is still in its infancy (e.g., Hanson, 2006; MacDorman, 2006). Possibly for this reason, almost no attention has been given to determining where along the DHL there is greater difficulty in *perceptual discrimination* (*PD*) (Looser and Wheatley, 2010; Cheetham et al., 2011) and to whether greater PD difficulty does relate to an increase in negative affective experience.

The UVH' prediction is based on the implicit assumption that PD difficulty is greatest at or near the point along the DHL at which there is greatest categorization ambiguity (i.e., the category boundary). There are two potential problems with this assumption. The first is that it conflicts with the general consensus that there is normally less PD difficulty at or near the category boundary compared with other regions of a perceptual dimension like the DHL (e.g., Harnad, 1987). The second is that PD difficulty might actually be most pronounced for categorically unambiguous human stimuli compared with other stimuli along the DHL. The potential impact of these two problems on the UVH is apparent in the following thought experiment. If we assume that PD difficulty is in fact attenuated at the category boundary and enhanced for human category exemplars (as tested in Experiment 1 of the present study) but that the UVH's prediction is otherwise correct (i.e., a positive relationship between PD difficulty and negative affect), it follows that greater negative affect should be experienced for objects and characters at the human category end of the DHL. This conclusion is, however, difficult to reconcile with the very idea that Mori sought to express in the UVH and more generally with reports of increased positive affect for human compared with nonhuman faces (e.g., Looser and Wheatley, 2010), unless it is assumed that the direction of the UVH' conjectured relationship between PD difficulty and affect is also incorrect (as tested in Experiment 2 of the present study).

These potential problems with the implicitly assumed distribution of PD difficulty along the DHL can be considered in terms of the literature on *categorical perception* (CP, for CP see e.g., Goldstone and Hendrickson, 2010; for the similar *perceptual magnet effect*, see Kuhl, 1991). CP refers to the phenomenon that the cognitive representation of *psychological similarity space* (such as along a perceptual dimension like the DHL) can be selectively deformed (Livingston et al., 1998). Deformation (or warping) of psychological similarity space is evident when, relative to a baseline of comparison, physical differences between stimuli within a category are subjectively perceived to be more similar (i.e., less discriminable) than equally spaced physical differences between stimuli from two different categories that are subjectively perceived to be less similar (i.e., more discriminable).

Many studies of CP have focused on facial processing. Studies of CP such as for famous faces (Beale and Keil, 1995), unfamiliar faces (Levin and Beale, 2000), facial expressions (Ectoff and Magee, 1992) and faces of different gender (Bülthoff and Newell, 2000) reveal a relatively symmetrical pattern of warping and PD performance along facial continua. This pattern is characterized by enhanced discriminability of stimuli (i.e., less PD difficulty) at the category boundary (rather than greater PD difficulty as assumed in the UVH) and similarly attenuated discriminability of stimuli (i.e., greater PD difficulty) within both categories. The similarly attenuated PD within the categories likely relates to the assumption in studies of CP (such as those in the preceding) that there is comparable (or symmetrical) category knowledge, categorization experience, and processing of continua endpoints from which morph continua are generated.

In contrast to this symmetry, the UVH was originally formulated on the basis of (informal) observation of individuals with extensive everyday experience processing human others but comparably little perceptual and categorization experience processing humanlike robotic characters. This implicit assumption in the UVH that categorization experience is asymmetrical for human compared with nonhuman others is also implicit in most uncanny-related studies (e.g., Yamada et al., 2013). These studies have typically examined participants who have everyday expertise in facial processing of human category exemplars (see Diamond and Carey, 1977; Tanaka, 2001) but, by virtue of the innovative nature of avatar and robot research and design and of the methods used to generate experimental stimuli, comparatively little if any such experience processing the subtle perceptual manipulations of and differences in human likeness between the nonhuman stimuli under investigation.

It is conceivable that differential experience in perceptual and category information processing will influence perceptual sensitivities and PD difficulty along the DHL. (Gibson, 1991; Hall, 1991; Goldstone, 1994; Harnad, 1987; Sigala et al., 2011). For example, compared with PD performance before training (using continua for which symmetrical knowledge of continua endpoints can be assumed), categorization experience with novel continua based on line drawings of fictitious animals (Livingston et al., 1998), with natural unfamiliar faces (Kikutani et al., 2008, 2010) and with faces of identical twins (Stevenage, 1998) is reflected in greater PD difficulty for within-category stimuli and lesser PD difficulty for the between category stimuli that straddle the category boundary. It would be consistent with such findings that asymmetry in categorization experience with human faces (for which there is everyday expertise due to a history of normal social interaction) compared with novel nonhuman faces (for which there is comparatively little or no such expertise) is reflected in a corresponding asymmetry in PD performance along the DHL. This would mean greater PD difficulty for within-category human stimuli compared with lesser PD difficulty for within-category nonhuman stimuli.

In the first of three experiments, we tested whether the distribution of PD difficulty along the DHL implicitly assumed in the UVH is correct. Considering the influence of categorization experience on CP and psychological similarity space reported in the preceding studies, we anticipated, firstly, that faces within the human category would generally be more difficult to discriminate compared with those closest to or at the category boundary. Second, we anticipated that faces within the human category would generally be more difficult to discriminate compared with those within the nonhuman category. To examine this, we delineated the profile of PD performance for morphed faces drawn from morph continua representing the DHL. The continua were generated from avatar (i.e., computer-generated characters) and human parent faces. The morphed faces were presented in an *ABX PD task* (Liberman et al., 1957; this task is described in detail in Section Design and procedure). Campbell et al. (1997) used the ABX task to investigate CP along other dimensions of human likeness and showed that this task is sensitive to differences in perceptual processing between human and nonhuman faces. Signal detection analysis was used to assess discrimination sensitivity. A *two-alternative forced choice categorization task* (described in detail in Section Design and procedure) was conducted after the ABX task in order to define the profile of categorisation ambiguity and the location of the category boundary along the continua. The second experiment replicated the findings of the first experiment. In the second experiment, we tested the UVH' predicted relationship between increased PD difficulty and negative affective experience. In the third experiment, we explored the possibility that the asymmetry in PD difficulty along the DHL reported in Experiments 1 and 2 might be attributable to a *differential processing bias*. This bias means that avatars might be processed at a category level and human faces at an exemplar level, this resulting in differences in PD performance between avatar and human faces of the DHL.

## Study 1: ABX perceptual discrimination and forced choice categorization tasks

### Materials and methods

#### Participants

Healthy adult volunteers (*N* = 49, 29 female, mean age 21.8 years; range 19–25 years) with no record of neurological or psychiatric illness and no current medication use were recruited for the study. All study participants were students of the University of Zurich, native or fluent speakers of Swiss or Standard German, and consistently right-handed, as assessed with self-rating scales (Annett, 1970). Each participant confirmed after completion of the experiment having had no previous experience designing or modifying computer-generated characters as for example in *virtual reality* (*VR*) role-playing games, second life, or VR environments or using such environments (e.g., for psychotherapy, rehabilitation, training, e-commerce or virtual reality-based research) and explicitly no previous experience (e.g., in video games) with the kind of highly humanlike characters and manipulations of human likeness presented in the current study. At debriefing, one participant reported uncertainty about the correct use of the response buttons and 3 others about the meaning of the label “avatar” in the forced choice categorization task. Analyses with and without the data of these four participants had no impact on the pattern of findings. The findings are reported on the basis of the complete data set. Written informed consent was obtained before participation according to the guidelines of the Declaration of Helsinki. Each volunteer received 20 Swiss Francs for participation. The study and all procedures and consent forms were approved by the Ethics Committee of the University of Zurich.

#### Stimuli

**Figure 2:**
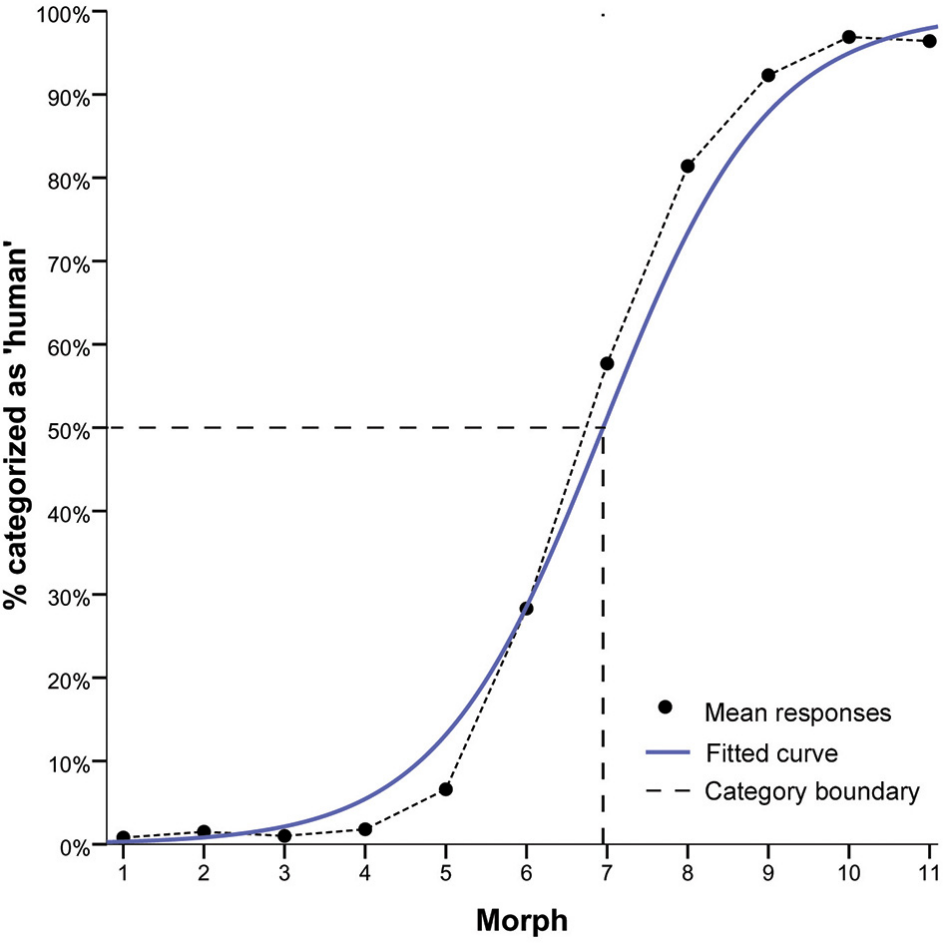
**Results of the forced choice classification task**. Mean responses are depicted in terms of % of “human” responses. The mean grand average across all continua (continuous blue line), fitted logistic curve based on the grand mean (black line), and the category boundary (dashed gray line) are shown. The category boundary indicates the point of maximum uncertainty of 50% in categorisation judgements along the continua. The logistic-shaped curve shows a lower and upper asymptote of avatar and human categorisation responses and a step-like response function consistent with the presence of a category boundary. Morph M7 shows the greatest categorisation ambiguity.

Morph continua were generated to represent the DHL, using the software Fantamorph® (Abrosoft http://www.abrosoft.com). These were produced in the same way as in previous studies (for an example continuum, see e.g., Cheetham et al., 2011). Eight color photographic images of natural faces and 8 color images of avatar faces were used as parent faces to produce 8 morph continua. The selection of continua was based on previous pilot testing to ensure like performance across continua (i.e., same morph position of the category boundary and shape of the response function). Each continuum comprised 11 different morphed images from the avatar endpoint (number 1) to the human endpoint (number 11), each morph being separated by an increment of 10% in physical difference (see Figure 2B). All parent faces were male, indistinctive, presented with full face, frontal view, direct gaze, neutral expression and no other salient features such as facial hair and jewelry. Avatars were generated with the modeling suite Poser 7® (Smith Micro Software, http://www.smithmicro.com) for detailed adjustment of facial geometry and texture (e.g., age and configural cues) to closely match the corresponding human face. Matching aimed to minimize perception of biological motion due to quick successive presentation of morphs (Schultz and Pilz, 2009) and to ensure perception of faces in the two-step procedure of the ABX as having the same identity. Adobe Photoshop 7.0® (http://www.adobe.com) was used for image editing. Before morphing, the external features of each parent face were masked with an elliptic form and black background (96 dpi and 560 × 650 pixels), and contrast levels, overall brightness and skin tone of the parent faces of each continuum were adjusted to match.

#### Design and procedure

All participants were tested individually by a research assistant blind to the purpose and hypotheses of the study. Following established procedure (Newell and Bulthoff, 2002), the PD ABX task was conducted first followed by the two-alternative forced-choice categorization task. The experiment lasted approximately 40 min, with a short break between the discrimination and categorization tasks.

***Perceptual discrimination ABX task***. The UVH does not suggest how DP difficulty should be operationalised and tested. For PD, the ABX discrimination task was used (Liberman et al., 1957; Harnad, 1987). This entails presentation of trials in which pairs of different face stimuli (A and B) are followed by a second presentation of either A or B as the target stimulus X. Participants are required to view all three images and respond by button press to indicate whether A or B is identical to (i.e., the same as) X. A 2-step discrimination procedure was applied so that stimulus B differed in physical distance along the continuum from stimulus A by two steps (i.e., 1–3, 2–4, 3–5, etc.). To counterbalance the sequence of face pairs, each pair was presented four times, once in each of the possible combinations (i.e., AB-A, BA-B, AB-B, BA-A). Both faces of each presented pair were always drawn from the same continuum in which they were originally morphed. The presentation of face pairs was pseudo-randomized so that no trails using face pairs from corresponding morph positions of other continua were presented in sequence.

Written instructions were presented on the screen before commencement of the experiment. Participants performed a pre-test of 5 trials (using stimuli drawn at random from continua that were not included in the main test) to ensure comprehension of the instructions and correct use of the response buttons. The background on the monitor was always black. Stimuli A and B were presented for 750 ms immediately followed by stimulus X, which remained on screen until the response was made or till time-out at 4 s. The inter-trail interval was 1500 ms. Response accuracy and *response time* (*RT*) were measured for each trial, including the practice trials.

The ABX task (and forced-choice classification task described in the next section) was conducted in a sound attenuated and light-dimmed room, and morph stimuli were presented on a LCD monitor (1280 × 1024 resolution, 60 Hz refresh rate), using Presentation® software (Version 14.1, www.neurobs.com). The stimuli (400 × 500 pixels) were presented at a viewing distance of 62 cm.

***Two-alternative forced-choice categorization task***. The same stimuli presented in the ABX task were presented in a two-alternative forced-choice categorization task. This task commenced with the presentation of written instructions. Subsequently, participants performed a practice pre-test of 5 trials, using the same stimuli used in the pre-trials of the ABX task. Having ensured task comprehension and correct use of the response buttons, the participant initiated testing by pressing a button. The forced-choice categorization task normally follows the PD task in order to minimize the potential influence of labeling on discrimination performance (Newell and Bulthoff, 2002). To minimize this further, the labels “avatar” and “human” were first used during task instruction for the forced choice task. The background on the monitor was always black. All morph stimuli were presented twice, individually, centrally, and in random order with the constraint that stimuli from corresponding morph positions of other continua were not presented in sequence. Each trial began with the presentation of a fixation point for 500 ms (participants were required to maintain fixation), followed by a morph image for 750 ms. The participant was asked to identify the stimulus quickly and accurately as either an avatar or human by pressing one of two response keys. A black screen with fixation point remained after presentation of the morph image until the participant pressed the response key, after which a blank black screen without fixation cross remained for 1500 ms until the next trial began.

All data analyses were performed using SPSS version 21.0 (http://www.ibm.com). MATLAB 2006b (http://www.mathworks.ch) was used to implement the Palamedes routines (Prins and Kingdom, 2009) for signal detection analysis of data from the ABX task.

### Results

The response data for avatar vs. human category judgments in the forced choice categorization task were analyzed (Section Forced choice categorization task: Responses, logistic function, and category boundary) to determine the choice of categorically ambiguous and unambiguous morphs for use in the analyses of PD performance (Section Forced choice categorization task: Response times).

#### Forced choice categorization task: responses, logistic function, and category boundary

The slope of the categorization response function was used to summarize the category judgments by fitting logistic function models to the data of each participant across continua. The parameter estimates derived from each model were entered in analyses of logistic function of categorization responses and of the category boundary.

For the logistic function of categorization responses, the parameter estimates were tested against zero in a one-sample *t*-test. The result showed a highly significant logistic component [*t*_(48)_ = 44.31, *p* > 0.001] consistent with the presence of a category boundary (Harnad, 1987) (see Figure 2).

To compute the value of the category boundary (i.e., *y* = 0.5: -ln[β 0]/ln[β 1]), the parameter estimates β 0 and β 1 of each participant's logistic function model were used. The mean category boundary value was *M* = 6.95. This value indicates the actual morph position along the continua that corresponds with the ordinate midpoint between the lower and upper asymptotes, that is, the point of maximum uncertainty of 50% in categorization judgments. Across continua, morph M7 is closest to this boundary (Figure 2; see also Supplemental Figures 1, 3, 5 for the results of the forced choice categorization task of each experiment with error bars).

**Figure 3:**
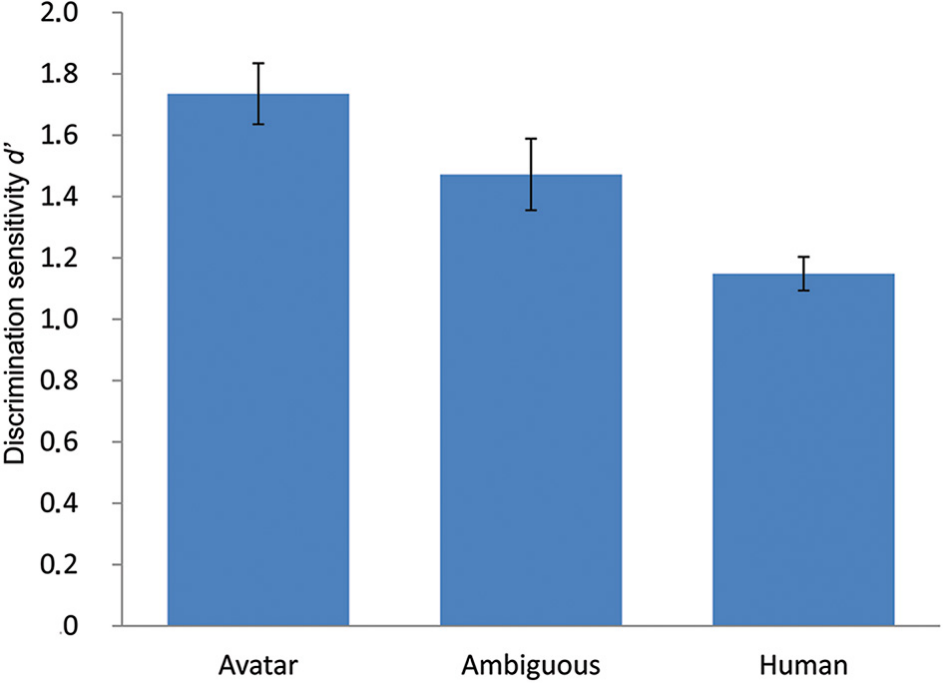
**Results of the ABX perceptual discrimination task**. The figure depicts mean discrimination sensitivity *d*′ in the ABX perceptual discrimination task for unambiguous avatar and human and highly ambiguous faces. The profile of *d*′ shows a marked asymmetry along the continua, meaning that unambiguous avatar (and the ambiguous faces) are perceived as more dissimilar than equally spaced human faces. Contrary to the implicit assumption in the UVH, perceptual discrimination difficulty is greatest for human faces. The error bars indicate 1 *SE* (*N* = 49).

To show this profile of high and low ambiguity in categorization judgments more clearly, we tested for differences in category decisions between the unambiguous avatar (i.e., M3, M4, M5) and human faces (i.e., M9, M10, M11) and the most ambiguous faces (i.e., M7). This choice of morphs permitted control for physical morph distance along continua between the ambiguous M7 and the unambiguous avatar and human faces. A one-way *repeated measures of analysis of variance* (*RM-ANOVA*) was performed on the dependent variable mean “categorization” response of each participant across continua, using the factor “morph” position (3 levels: “M3, M4, M5,” “M7,” “M9, M10, M11”). Greenhouse-Geisser adjustment was applied to correct the degrees of freedom for violation of the sphericity assumption (and applied as appropriate in all subsequent analyses). This analysis showed a highly significant effect for morph position, *F*_(1.27, 58.93)_ = 455.26, *p* < 0.001. Mean categorization difficulty for M7 was *M* = 0.58 (*SE* = 0.04), while that for the human faces was *M* = 94.52 (*SE* = 0.01) and for avatar faces *M* = 4.02 (*SE* = 0.01) (see Figure 3).

#### Forced choice categorization task: response times

Differences in category ambiguity, as indicated by the logistic-shaped response function, are likely to be reflected in different *RT* for category judgments. Before data analysis, short RT latencies of less than 100 ms were excluded. RT data for long latency outliers were screened by z-standardizing and filtering out data points using *z* = 3 as a cut-off score (Van Selst and Jolicoeur, 1994). Analyses were conducted with and without outliers. These analyses produced the same pattern of results. The findings are therefore reported for the complete data set. Confirming RT differences in category decision difficulty, a one-way RM-ANOVA with morph position (11 levels: M1-M11) and RT as the dependent variable showed a main effect for morph position, *F*_(4.58, 215.09)_ = 41.23, *p* < 0.001.

The longest response latencies would be expected to correspond with the morph position closest to the category boundary, that is, at M7 (see Supplemental Figure 2). But inspection of Supplemental Figure 2 indicates that RT for M6 and M7 are similarly long. The tests of planned within-subject contrasts in the preceding analysis showed no significant difference between M6 and M7 in RT. Given that M7 and the category boundary are so closely aligned, the following analysis compared ambiguity at M7 with the unambiguous avatar (i.e., M3, M4, M5) and human faces (i.e., M9, M10, M11), but a re-run of the same analysis using the aggregate mean of M6 and M7 instead of just M7 produced the same pattern of results. A one-way RM-ANOVA analysis with “morph” positions (3 levels: “M3, M4, M5,” “M7,” “M9, M10, M11”) and RT in ms as dependent variable was conducted. The analysis showed a highly significant effect for morph position [*F*_(2.96, 58.93)_ = 45.22, *p* < 0.001]. Pre-planned contrasts showed that RT was longer significantly longer for human (*M* = 1073, *SE* = 44) than for avatar faces (*M* = 898, *SE* = 33), *F*_(1, 48)_ = 15.72, *p* > 0.001, and that RT for M7 (*M* = 1348, *SD* = 60) differed highly significantly from RT for the other avatar and human morph positions (*M* = 928, *SD* = 0.19), *F*_(1, 48)_ = 67.49, *p* < 0.001.

#### ABX perceptual discrimination task

Differences in the ability to perceptually discriminate between pairs of morphs (M6-M8) straddling the ambiguous M7 and between pairs of unambiguous morphs within the avatar (M3-M5, M4-M6) and human (M8-M10, M9-M11) face categories were tested. This choice of avatar and human morph pairs ensured control for the physical morph distance along the continua between the ambiguous and unambiguous faces. The mean value of PD was compared in a one-way RM-ANOVA with factor morph position (3 levels: “M3-M5, M4-M6,” “M6-M8,” “M8-M10, M9-M11”) using *d*′ as dependent variable (Best et al., 1981). *d*′ is used a measure of discrimination performance derived from Signal Detection Theory (e.g., Macmillan and Creelman, 2005) that takes effects of response bias (*c*) into account. This measure is used instead of the percentage of correct different responses to different pairs (Francis and Ciocca, 2003). A differencing model was applied to compute *d*′ because this is considered to best reflect the decision strategy used in the ABX task (Pierce and Gilbert, 1958; Hautus and Meng, 2001; Macmillan and Creelman, 2005).

This analysis showed a significant effect for morph pair position, *F*_(2, 96)_ = 14.68, *p* < 0.001. Tests of planned within-subject contrasts showed that PD of faces within the avatar category (*M* = 1.74, *SE* = 0.1) was significantly greater than that of ambiguous faces at the category boundary (*M* = 1.47, *SE* = 0.12) [*F*_(1,48)_ = 5.59, *p* = 0.022] and of faces within the human category (*M* = 1.15, *SE* = 0.05), *F*_(1, 48)_ = 38.54, *p* < 0.001. PD of ambiguous faces was significantly greater than that of faces within the human category, *F*_(1, 48)_ = 7.5, *p* = 0.009.

A one-way RM-ANOVA with “morph position” (11 levels) and *c* as the dependent variable for response bias showed no significant differences for *c*.

### Discussion

The data confirm that there are differences in PD difficulty as a function of human likeness along the DHL. But the pattern of PD is entirely different than that implicitly assumed in the UVH. Firstly, and as expected on the basis of previous studies of CP, PD of faces at the category boundary is enhanced compared with PD of within-category human faces. Second, PD of within-category avatars is also enhanced compared with PD of within-category human faces, thus supporting the suggestion that PD performance along the DHL might be asymmetrical.

Given that the UVH predicts enhanced negative affective experience as a function of enhanced PD difficulty, these findings would mean—assuming that the UVH is otherwise correct—that human faces should evoke more negative affect compared with ambiguous faces and unambiguous avatar faces. This is clearly inconsistent with the idea that Mori sought to convey in his graphical representation of his hypothesis, and the available evidence from uncanny-related research suggests that enhanced feelings of strangeness for human category exemplars is highly unlikely. Self-ratings of comparably well-controlled morph continua show that positive ratings (e.g., pleasantness) increase with greater human likeness (e.g., Looser and Wheatley, 2010).

In a second experiment, we tested whether there is nevertheless evidence in favor of the UVH' prediction that enhanced PD difficulty is associated with greater negative affective experience. The UVH conceptualizes affective experience as *shinwakan*, an ambiguous Japanese neologism that Mori used to describe the positive and negative character of affective experience of humanlike objects. There have been various renderings of shinwakan's meaning in uncanny-related research, including comfort level, familiarity, eeriness, pleasantness, likability, empathy and affinity (e.g., MacDorman and Ishiguro, 2006; Bartneck et al., 2007; Seyama and Nagayama, 2007; Green et al., 2008; Tinwell et al., 2011; Dill et al., 2012; Mori, 2012; MacDorman et al., 2013; Burleigh et al., 2013; see also Ho and MacDorman, 2010). To examine affective experience, we used an *ad hoc* self-rating scale based on the UVH' bi-polar dimension of *familiarity* (i.e., feelings of familiarity vs. strangeness). Familiarity was selected because this rendering of shinwakan has been used frequently in research, it is most often used to denote the affective dimension of the UVH in its illustration (see Figure 1), and because it arguably best captures the apparent meaning of shinwakan that Mori sought to convey in the UVH's description. Clearly, there are alternative approaches to examining affective experience of human like objects and characters based on well-validated dimensions of affective experience and measures of these. The aim of this experiment was to test affective experience as conceptualized in the UVH in relation to PD difficulty.

## Experiment 2

The materials, methods and analyses in Experiment 2 were identical to those in Experiment 1, with two exceptions. Firstly, the presented morphs were drawn from continua that were generated anew. This was done by switching the source image (i.e., avatar) and destination image (i.e., human) for morphing in Experiment 1 so that the human was now the source and the avatar the destination image. The continua were then re-morphed, and the morphs were labeled M1 (avatar) to M11 (human) as in Experiment 1. The reason for switching the source and destination images and of re-morphing the stimuli was to exclude the possibility that the strong asymmetry in PD performance in Experiment 1 was simply a systematic artifact of any nonlinearity in the morphing algorithm used to generate the continua. If it was a systematic artifact, the PD data in Experiment 2 would show a similarly skewed pattern of PD along the DHL, with however enhanced PD for the human instead of the avatar faces. Second, participants performed the ABX task followed this time by the self-rating task, in which to report feelings of familiarity, and only then by the two-alternative forced choice categorization task. The latter task was performed last to ensure that any effects in ratings were not biased by explicit processing of faces for forced categorization.

The UVH does not suggest how DP difficulty and feelings of familiarity should be operationalised and tested. We used our measure of discrimination sensitivity *d*′ to indicate DP performance, as applied in Experiment 1, and, in keeping with the favored approach to date in uncanny research, we used subjective ratings to indicate feelings of familiarity in the self-rating task. The task requirements, instructions and stimulus presentation conditions of the self-rating task were identical to those described for the two-alternative forced choice categorization task in Experiment 1, with the exception that participants viewed and rated the subjective feeling of familiarity evoked by each morphed stimulus on a 5-point Likert scale. The scale ranged from very strange (1) to very familiar (5). To test the relationship between DP difficulty and feelings of familiarity we took an inter-individual differences approach. We tested whether individual variability in the ability to discriminate between a pair of morphed faces (e.g., M2-M4) predicts individual variability in self-rated feelings of familiarity for the face (e.g., M3) that the given face pair straddles. This approach assumes that there are stable individual differences in the relationship between familiarity ratings and discrimination performance. If Mori's prediction is correct, greater PD difficulty should be associated with increased feelings of strangeness (i.e., with less familiarity). This was tested.

### Participants

A new sample of *N* = 49 volunteers (34 female, mean age 21.9 years; range 19–31 years) not involved in Experiment 1 participated in Experiment 2.

### Results

#### Forced choice categorization task: responses, logistic function, and category boundary

**Figure 4:**
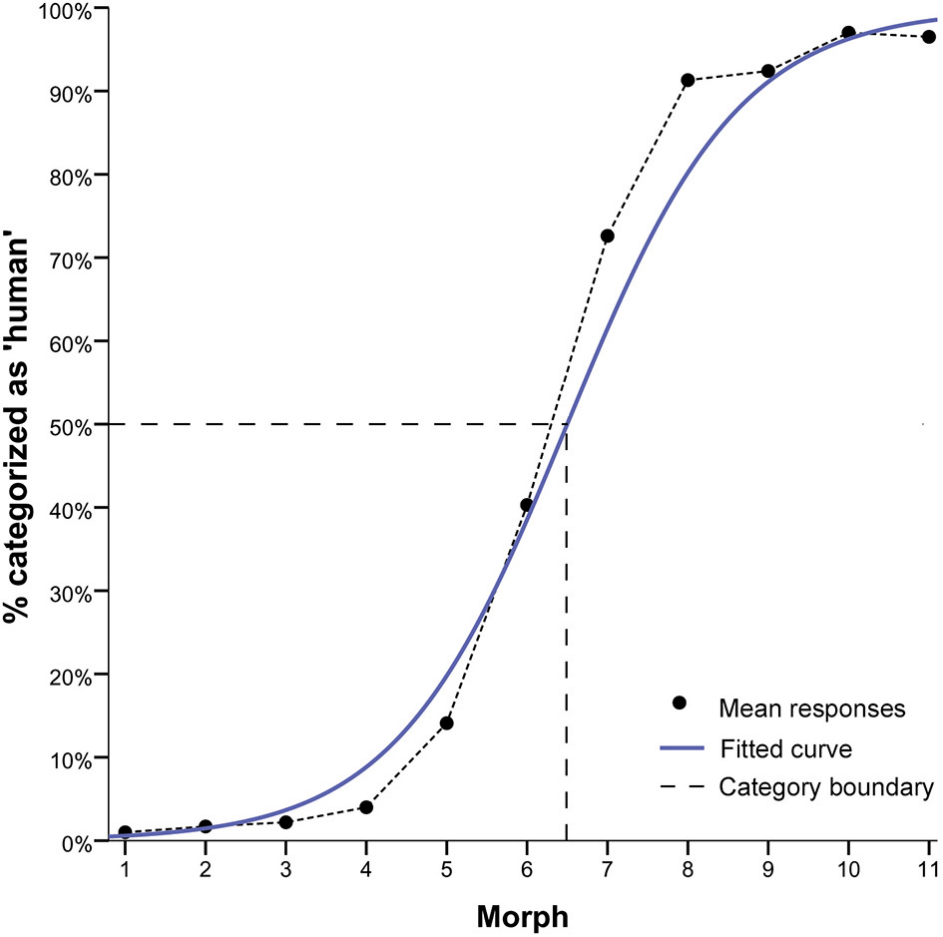
**Results of the forced choice classification task**. Mean responses are depicted in terms of % of “human” responses, with the grand average across all continua (continuous blue line), the fitted logistic curve based on the grand mean (black line), and the category boundary (dashed gray line) to indicate the point of maximum uncertainty of 50% in categorisation judgements along the continua. Results indicate a step-like response function consistent with the presence of a category boundary. Morph M6 shows the greatest categorisation ambiguity.

The parameter estimates derived from each logistic function model of each participant across continua were tested against zero in a one-sample *t*-test and showed, as in Experiment 1, a highly significant logistic component [*t*_(48)_ = 27.83, *p* > 0.001] (see Figure 4). Based on the parameter estimates β 0 and β 1, the mean category boundary value was *M* = 6.6. Across continua, the most ambiguous face morph M6 is closest to this boundary.

To show the effects of this profile of high and low ambiguity in categorization judgments more clearly, we tested for differences in category decisions between the unambiguous avatar (i.e., M2, M3, M4) and human faces (i.e., M8, M9, M10) and the most ambiguous faces (i.e., M6). Consistent with the approach in Experiment 1, the choice of morphs permitted control for physical morph distance along continua between M6 at the category boundary and the avatar and human faces. A one-way *RM-ANOVA* was performed on the dependent variable mean “categorization” response of each participant across continua, using the factor “morph” position (3 levels: “M2, M3, M4,” “M6,” “M8, M9, M10”). This analysis showed a highly significant effect for morph position [*F*_(1.22, 58.52)_ = 483.72, *p* < 0.001]. Categorization difficulty for M6 was closest to chance level of 50% (*M* = 40.31; *SE*= 3.73), while that for the human faces was *M* = 93.58 (*SE* = 1.13) and for avatar faces *M* = 2.63 (*SE* = 0.44) (see Figure 4).

#### Forced choice categorization task: response times

We verified whether differences in category ambiguity are reflected in the RT for category judgments. Data were screened for outliers as in Experiment 1 and analyses conducted with and without these. These analyses produced the same pattern of results for which reason the findings for the complete data set are reported. Confirming RT differences in category decision difficulty, a one-way RM-ANOVA with morph position (11 levels: M1-M11) and RT as the dependent variable showed a main effect for morph position, *F*_(4.58, 220.22)_ = 39.03, *p* < 0.001.

Inspection of the RT data (see Supplemental Figure 4) indicates that the longest response latencies correspond with the most ambiguous morph M6. A one-way RM-ANOVA analysis with “morph” positions (3 levels: “M2, M3, M4,” “M6,” “M8, M9, M10”) and RT in ms as dependent variable was conducted. The analysis showed a highly significant effect for morph position, *F*_(2, 96)_ = 54.99, *p* < 0.001. Pre-planned contrasts showed that RT was longer significantly longer for human (*M* = 957, *SE* = 34) than for avatar faces (*M* = 751, *SE* = 23), *F*_(1, 48)_ = 15.72, *p* > 0.001, and that RT for M6 (*M* = 1191, *SD* = 53) differed highly significantly from RT for the other morph positions (*M* = 851, *SD* = 0.37), *F*_(1, 48)_ = 65.91, *p* < 0.001.

#### ABX perceptual discrimination task

An independent samples *t*-test (Experiment 1 vs. Experiment 2) using *d*′ for each morph pair position in the ABX task (i.e., pairs M1-M3 through to M9-M11) of each participant across continua as dependent variable showed that discrimination performance for each morph pair was not significantly different between Experiments 1 and 2. The following results indicate also that the PD effects in Experiment 1 are comparable to those in Experiment 2.

Given that face morph position M6 was closest to the category boundary in Experiment 2, differences in the ability to perceptually discriminate between pairs of morphs (M5-M7) straddling the *ambiguous* M6 was compared with the ability to perceptually discriminate between unambiguous morphs within the *avatar* (M1-M3, M2-M4, M3-M5) and *human* (M7-M9, M8-M10, M9-M11) face categories. These morph pairs were selected because they straddle the morph positions M2, M3, M4, M6, M8, M9, M10 that were analyzed in the forced choice task of Experiment 2 and because this choice of pairs ensures control for physical morph distance between the ambiguous and the unambiguous human and avatar faces. The mean value of discrimination sensitivity was compared in a one-way RM-ANOVA with factor morph position (3 levels: “M1-M3, M2-M4, M3-M5,” “M5-M7,” “M7-M9, M8-M10, M9-M11”) using *d*′ as dependent variable.

**Figure 5:**
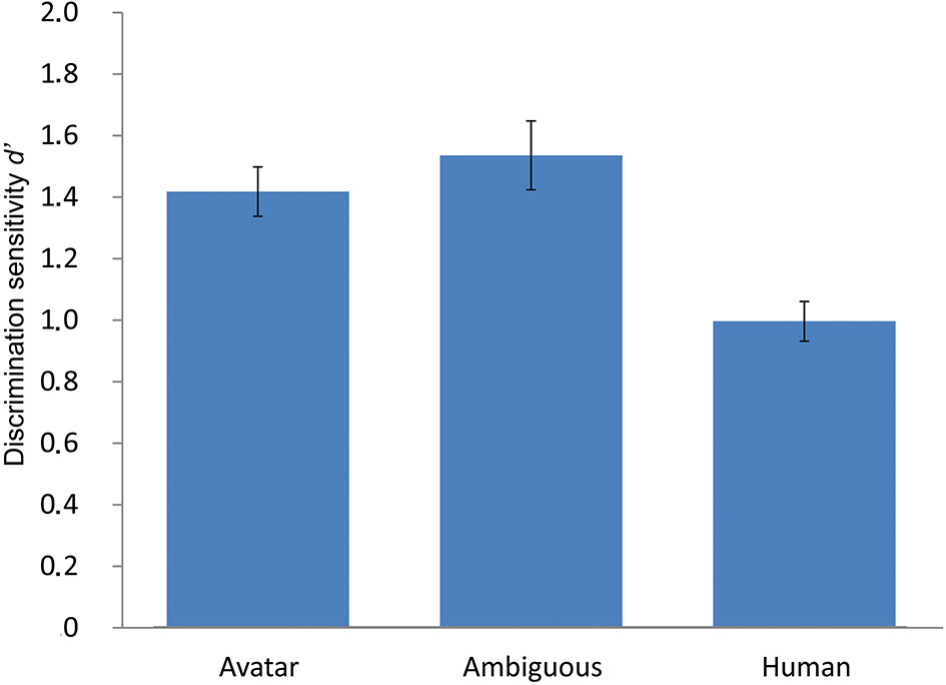
**Results of the ABX perceptual discrimination task**. The figure depicts mean discrimination sensitivity *d*′ in the ABX perceptual discrimination task for unambiguous avatar and human and highly ambiguously faces. The profile of discrimination sensitivity replicates that found in the first experiment. Perceptual discrimination difficulty is greatest for human faces. The error bars indicate 1 SE (*N* = 49).

This analysis showed a significant effect for morph pair position, *F*_(2, 96)_ = 16.52, *p* < 0.001 (see, Figure 5). Tests of planned within-subject contrasts showed that discrimination of avatar faces (*M* = 1.41, *SE* = 0.08) was significantly greater than that of faces within the human category (*M* = 0.99, *SE* = 0.07), *F*_(1, 48)_ = 27.59, *p* > 0.001. Discrimination of ambiguous faces at the category boundary (*M* = 1.53, *SE* = 0.11) was not significantly greater than that of faces within the avatar category (*M* = 1.41, *SE* = 0.08) [*F*_(1, 48)_ = 1.11, *p* = 0.299], but it was significantly greater than that of faces within the human category (*M* = 0.99, *SE* = 0.07), *F*_(1, 48)_ = 25.76, *p* < 0.001.

It should be noted that the most ambiguous morph was M7 in Experiment 1 and M6 in Experiment 2. This means that the choice of morph pairs for inclusion in the analyses of *d*′ in Experiment 1 is partially different than the choice in Experiment 2. To compare Experiments 1 and 2, the one-way RM-ANOVA in Experiment 2 was re-run, using this time the same morph positions selected in Experiment 1, that is, M3-M5 and M4-M6 for avatar faces, M6-M8 for the ambiguous M7, and M8-M10 and M9-M11 for human faces. This analysis showed the same pattern of significant effects for morph pair position [*F*_(2, 96)_ = 21.42, *p* < 0.001] and for the tests of planned within-subject contrasts (see Supplemental Figure 6). The contrasts showed that PD of faces within the avatar category (*M* = 1.67, *SE* = 0.11) was significantly greater than that of ambiguous faces at the category boundary (*M* = 1.34, *SE* = 0.11) [*F*_(1,48)_ = 12.87, *p* = 0.001] and of faces within the human category (*M* = 0.98, *SE* = 0.07), *F*_(1, 48)_ = 35.48, *p* < 0.001. PD of ambiguous faces was significantly greater than for faces within the human category, *F*_(1, 48)_ = 11.26, *p* = 0.002. Taken together, these analyses are consistent in indicating asymmetry in discrimination performance along the continua.

A one-way RM ANOVA with “morph position” (11 levels) and *c* as the dependent variable for response bias showed no significant differences for *c*.

### Familiarity ratings

**Figure 6:**
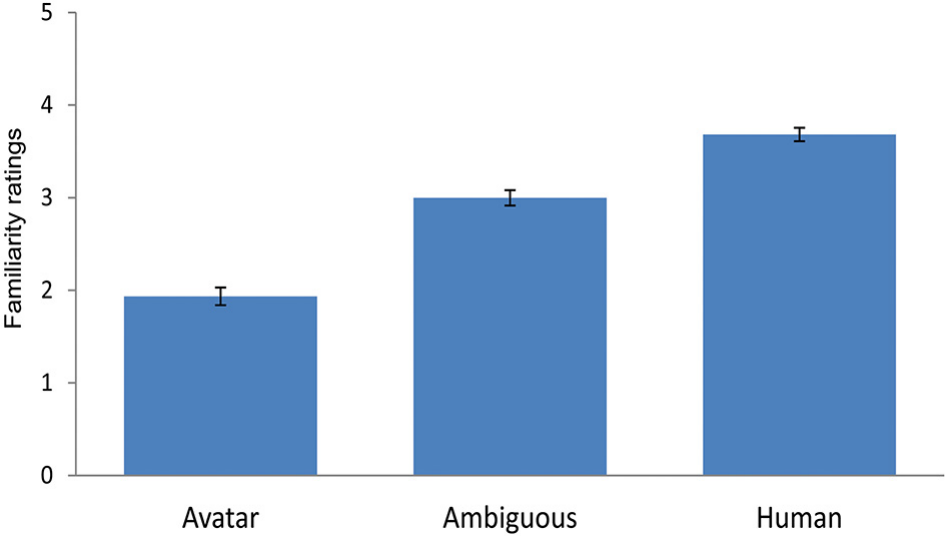
**Results of self-rating task for familiarity**. Overall, the figure illustrates a general increase in self-rated feelings of strangeness with decreasing human likeness from the human end of the continua. There is no indication of the uncanny effect predicted in the UVH. The error bars indicate 1 *SE* (*N* = 49).

Differences in mean familiarity ratings between the *unambiguous* avatar (i.e., M2, M3, M4) and *human* faces (i.e., M8, M9, M10) and the most *ambiguous* faces (i.e., M6) were tested using the same morph positions as in the analysis of the forced choice categorization task in Experiment 2 (Section Forced choice categorization task: Responses, logistic function, and category boundary). A one-way RM-ANOVA with the factor *morph* position (3 levels: “M2, M3, M4,” “M6,” “M8, M9, M10”) and the dependent variable *familiarity* rating of each participant across continua revealed a highly significant effect of morph position, *F*_(1.48, 70.93)_ = 180.61, *p* ≤ 0.001 (see Figure 6). Pre-planned contrasts showed a significant difference between the avatar morphs (*M* = 1.93; *SE* = 0.1) and M6 (*M* = 3; *SE* = 0.08) *F*_(1, 48)_ = 278.67, *p* ≤ 0.001 and between M6 and the human morphs (*M* = 3.68; *SE* = 0.07), *F*_(1, 48)_ = 53.02, *p* ≤ 0.001, and between the avatar and human morphs, *F*_(1, 48)_ = 27.59, *p* ≤ 0.001. Taken together, the data indicate that familiarity ratings increase negatively (i.e., greater strangeness) across the three stimulus conditions with increasing distance from the human end of the continua. This lends no support to the UVH' predicted increase in negative evaluations for the most ambiguous faces.

### Relationship between perceptual discrimination and familiarity ratings

The UVH predicts a positive relationship between greater PD difficulty and greater subjective experience of strangeness. To test this we examined whether individual variability in PD performance for face pairs predicts individual variability in ratings of subjective experience for the faces that the face pairs straddle. Pearson product-moment correlations were conducted using the mean data of each participant across continua of each *morph* in the familiarity rating task (i.e., “M2, M3, M4” for avatar, M6 for ambiguous, and “M8, M9, M10” for human faces) and the morph pairs that straddled these faces in the ABX task (i.e., “M1-M3, M2-M4, M3-M5” for *avatar*, M5-M7 for ambiguous, M7-M9, M8-M10, M9-M11 for human faces). Outlier detection was performed before analysis by means of boxplots. This indicated 1 outlier. After removal of this outlier, the analyses showed a highly significant (two-sided) negative correlation between PD performance and familiarity ratings for avatar faces [*r*_(48)_ = −0.314, *p* = 0.03] and for ambiguous faces [*r*_(48)_ = −0.494, *p* > 0.001]. There was no significant relationship between PD performance and familiarity ratings for human faces [*r*_(49)_ = 0.088, *p* = 0.533].

### Discussion

The data of Experiment 2 replicated those of Experiment 1 by showing the same pattern of PD asymmetry, that is, enhanced PD for highly ambiguous faces and highly unambiguous nonhuman faces but attenuated discrimination for highly unambiguous human faces. Based on a new sample of participants and re-morphed continua, this pattern re-affirms that the implicit assumption in the UVH, that is, greater PD difficulty in the categorically most ambiguous region of the DHL, is incorrect. It is in this region that the UVH suggests stronger feelings of strangeness compared with those evoked by neighboring less ambiguous human or humanlike stimuli. But the data show that greater feelings of strangeness are actually reported for the least human faces, and that feelings of strangeness diminish with increasing human likeness of the facial morphs.

While there is no indication of an uncanny effect as described in the UVH, these data are based on group averaging of data. It is however possible that there are inter-individual differences in the relationship between familiarity and PD difficulty that are concealed by data averaging and that these differences might reveal an effect consistent with Mori's suggestion. In fact, the correlative data show a significant relationship between PD difficulty and feelings of familiarity, but the direction of this relationship is the opposite of that predicted in the UVH. Increasing PD difficulty is associated with more positive feelings of familiarity. Interestingly, this effect only applies for nonhuman and ambiguous faces. There was no significant relationship between PD difficulty and familiarity for human faces. Critically, this correlative effect was greatest for ambiguous faces. Taken together, the correlative data suggest, irrespective of the question of the causal direction, that the UVH' prediction is most likely to be wrong.

The reason for asymmetry in PD performance along the continua is not clear. One potential explanation draws on the suggestion that human observers preferentially code other members of the human in-group (e.g., our human exemplars) differently than members of a nonhuman out-group (e.g., our highly humanlike avatars) (Cheetham et al., 2013; see the *other-race hypothesis*, Levin, 2000; *differential processing hypothesis*, Ostrom et al., 1993; *other-race effect*, Rhodes et al., 2006). This bias in coding means that individuals are tuned by categorization experience to detect subtle differences between other human individuals, thus facilitating face recognition among in-group members at the (individuating) exemplar level (see the *feature-selection hypothesis*, Levin, 2000). In contrast, individuals code information in the out-group that is more relevant for detection of out-group members, that is, information at the category level. At the category level, the best cognitive processing strategy for discriminating faces would be to code information indicating differences in human likeness along the DHL, thus enhancing discrimination of out-group members (i.e., our avatars). In contrast, a processing strategy that is more suited to face recognition of the individual human category exemplars than processing differences in human likeness along the DHL is more likely to result in poorer discrimination performance for human faces.

Face recognition among in-group members at the individuating level is more likely to rely on the use of configural information (Maurer et al., 2002), whereas there is evidence of less configural coding of out-group members (e.g., Rhodes et al., 1989; Fallshore and Schooler, 1995). Configural information relates to the individual arrangement of first- and second-order (e.g., nose-mouth distance) spatial relations among facial features (Rhodes, 1988). Configural processing is disrupted when faces are inverted instead of being presented upright (Diamond and Carey, 1986; Bartlett and Searcy, 1993; Rhodes et al., 1993; Rossion, 2009). If the asymmetry in PD between the avatar and human faces is attributable to a greater tendency to individuate human category exemplars than avatar category exemplars and a bias therefore toward greater configural processing of human exemplars, face inversion should reduce or eliminate the asymmetry. If on the other hand the asymmetry is not attributable to differences in configural processing, face inversion will have no impact on it. Experiment 3 was performed in order to test this.

## Experiment 3

The ABX and forced choice categorization tasks were performed. The task requirements, instructions and stimulus presentation conditions for these tasks were identical to those described for the two preceding experiments, with one exception. The re-morphed stimuli that were presented in Experiment 2 were inverted by rotating them 180°.

### Participants

A new sample of *N* = 25 volunteers (21 female, mean age 21 years; range 18–26 years) not involved in Experiments 1 or 2 participated in Experiment 3.

### Forced choice categorization task: logistic function, and category boundary

**Figure 7:**
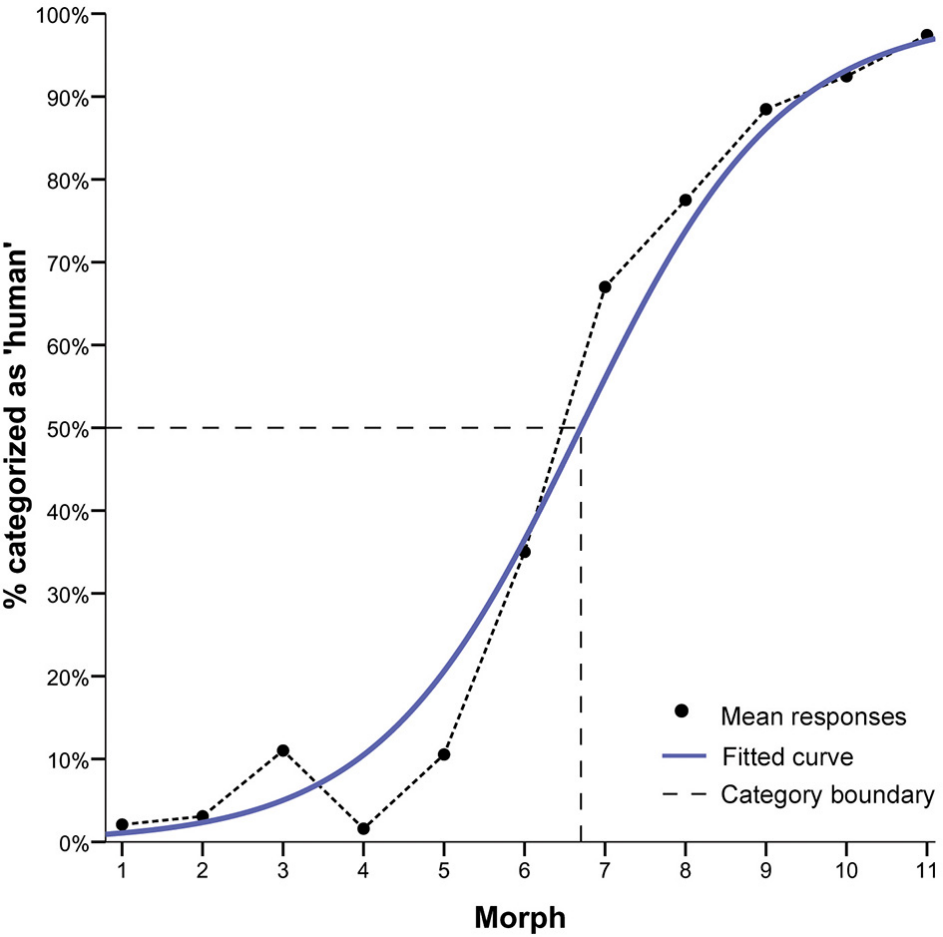
**Results of the forced choice classification task for inverted faces**. Mean responses are depicted in terms of % of “human” responses, with the grand average across all continua (continuous blue line), the fitted logistic curve based on the grand mean (black line), and the category boundary (dashed gray line) to indicate the point of maximum uncertainty of 50% in categorisation judgements along the continua. Results indicate a step-like response function consistent with the presence of a category boundary.

The parameter estimates derived from each logistic function model of each participant across continua were tested against zero in a one-sample *t*-test and showed, as in Experiments 1 and 2, a highly significant logistic component [*t*_(24)_ = 22.29, *p* > 0.001] (see Figure 7). Based on the parameter estimates β 0 and β 1, the mean category boundary value was *M* = 6.7. Across continua, the data show that the most ambiguous face morph M6 is closest to this boundary (Figure 7).

For completeness, the other analyses for the forced choice categorization task conducted in Experiments 1 and 2 (i.e., categorization responses and RT) were repeated for Experiment 3. These produced the same pattern of results as Experiments 1 and 2 and are reported together with Figures in the *Supplemental information Experiment 3*.

### Results: ABX perceptual discrimination task

An independent two-sample *t*-test (Experiment 3 vs. Experiment 2) using mean *d*′ of each participant across continua as dependent variable was conducted to compare PD performance in Experiments 2 and 3; these were compared because these experiments used the same re-morphed continua. This analysis showed that discrimination performance for each of the 9 morph pairs (i.e., M1-M3 to M9-M11) was not significantly different between Experiments 2 and 3. Levene's test of equality of variances indicated that the group variances for each of the 9 morph pairs could be treated as equal. For completeness, the same analysis was repeated to test for differences between Experiment 3 vs. Experiment 1. This showed a significant difference in discrimination between morph pairs M6-M8 [*t*_(72)_ = 2.12, *p* > 0.038] (note that M7 in Experiment 1 and M6 in Experiment 3 were the most ambiguous) and between the most human morph pairs M9-M11, [*t*_(72)_ = 3.5, *p* > 0.001]. There were no other differences (for the results of the three ABX experiments, showing all 9 morph pairs, see Supplemental Figure 7).

**Figure 8:**
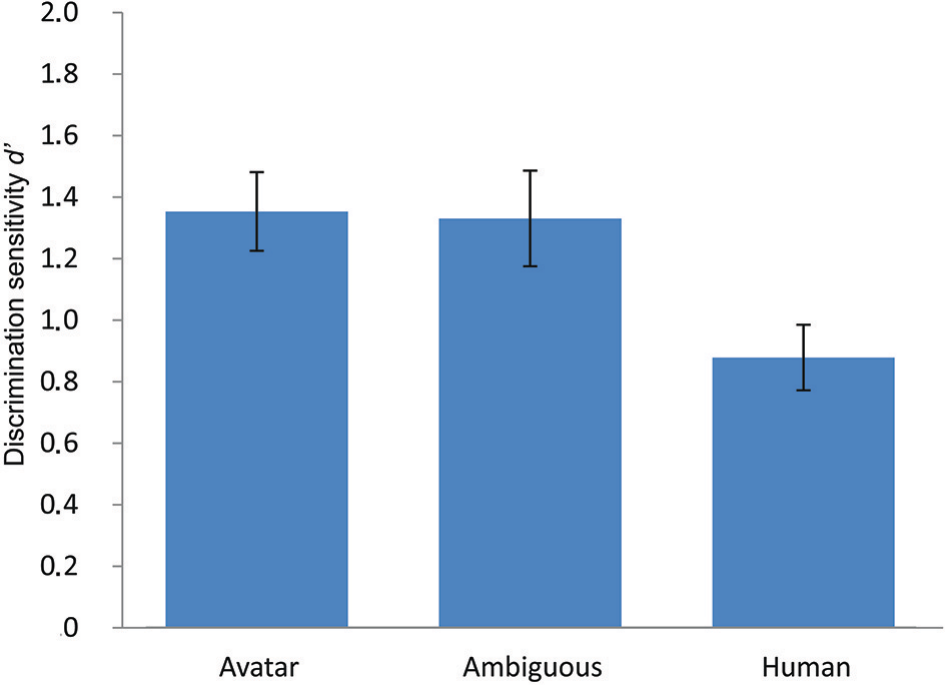
**Results of the ABX perceptual discrimination task for inverted faces**. This figure depicts mean discrimination sensitivity *d*′ in the ABX perceptual discrimination task for inverted unambiguous avatar and human and highly ambiguously faces. The data replicate those of experiments 1 and 2, showing the same asymmetry in perceptual discrimination performance along the dimension of human likeness. Face inversion had no impact on this, indicating that this asymmetry is not attributable to a differential processing strategy in which avatars are coded at a category and human faces at an individual level. The error bars indicate 1 *SE* (*N* = 25).

PD performance in Experiment 3 was then tested. Given that face morph position M6 was closest to the category boundary, differences in the ability to perceptually discriminate between pairs of morphs (M5–M7) straddling the *ambiguous* M6 compared with the ability to perceptually discriminate between unambiguous morphs within the *avatar* (M1-M3, M2-M4, M3-M5) and *human* (M7-M9, M8-M10, M9-M11) face categories were tested. This choice of morph pairs was based on the preceding data of the forced choice task, and ensured control for the physical morph distance between the ambiguous and unambiguous faces. The mean value of discrimination sensitivity was compared in a one-way RM-ANOVA with factor morph position (3 levels: avatar, ambiguous, human) using *d*′ as dependent variable (see Figure 8). This analysis showed a significant effect for morph pair position, *F*_(2, 48)_ = 11.18, *p* < 0.001.

Tests of planned within-subject contrasts showed the same pattern of significant differences in PD as in Experiment 2. PD of avatar faces (*M* = 1.44, *SE* = 0.13) was significantly greater than that of faces within the human category (*M* = 0.87, *SE* = 0.11), *F*_(1, 48)_ = 27.31, *p* > 0.001. As in Experiment 2, discrimination sensitivity for ambiguous faces at the category boundary (*M* = 1.32, *SE* = 0.15) was not significantly greater than for faces within the avatar category, F_(1, 24)_ = 1.03, *p* = 0.319, but it was significantly greater than for faces within the human category, F_(1, 24)_ = 9.5, *p* = 0.005.

The data thus indicate that face inversion had no differential impact on the ability to discriminate between faces along the continua.

A one-way RM ANOVA with “morph position” (11 levels) and *c* as the dependent variable for response bias showed no significant differences for *c*.

### Discussion

Experiment 3 explored the possibility that the asymmetry in PD reported in Experiments 1 and 2 might be attributable to a *differential processing bias*. This bias suggests that participants preferentially code human-category exemplars at the individual level and avatar-category exemplars at the category level. The data show that the inversion of faces had no impact on the asymmetry in PD, indicating that the asymmetry is not likely to be attributable to differences in configural coding and to a tendency to preferentially process human compared with avatar faces at an individual level.

## General discussion

The UVH conceptualizes the DHL as a linear dimension of physical similarity space. This space is considered to span between points within a nonhuman category representing similar objects or characters of various degrees of human likeness and a single point representing the human category (Figure 1). The problem with this conceptualization and, more importantly, its faithful application in uncanny studies and theoretical considerations (e.g., Ramey, 2005; Tinwell et al., 2011) is that it implicitly assumes that this space does not vary within the human category. The assignment of physically different morphs to the human category in the forced choice categorization task clearly shows that this assumption is wrong (see also e.g., Looser and Wheatley, 2010; Cheetham et al., 2011; Yamada et al., 2013).

The advantage of considering the human end of the DHL is that it provides a basis of comparison for understanding how other objects and characters along the DHL are perceived and experienced. This approach is important for the present study. The UVH predicts enhanced negative affective experience as a function of enhanced PD difficulty and suggests that this effect occurs at the point along the DHL at which categorization ambiguity is greatest. The data of the first and second experiments confirmed that there are differences in PD performance as a function of human likeness. But the pattern of differences in PD is very different than that implicitly assumed in the UVH. Firstly, and as expected on the basis of previous studies of CP, PD of faces at the category boundary is enhanced compared with PD of within-category human faces. Second, PD of within-category avatars is also enhanced compared with PD of within-category human faces. Together, these findings support the suggested asymmetry in PD along the DHL. In contrast to the UVH, they show that PD difficulty is greatest for human faces.

This finding of enhanced PD difficulty on the human side of the DHL's category boundary is reflected in the warped profile of psychological similarity space that is typically described for CP. This profile is characterized by attenuated PD performance for faces within the human category compared with enhanced PD performance for faces close to and at the category boundary (e.g., Livingston et al., 1998). In the present study, warping likely reflects the impact of perceptual and category learning processes over a person's history of everyday social interactive behavior with other members of the human category: All participants expressly reported no previous experience with our specific avatar parent faces, no previous experience with similarly humanlike faces (and robots), and no knowledge of previous experience with human likeness-related manipulations of perceptual features such as those applied along our morph continua. In contrast, they considered the human parent faces to be of the kind that they might typically encounter in normal everyday situations.

The impact of perceptual and category learning processes is that these likely lead to perceptual desensitization to within-category human features that are therefore perceived as more alike or *equivalent* and to enhanced perceptual sensitivity close to and at the category boundary to those stimulus features that facilitate assignment of category membership in everyday tasks (e.g., human vs. nonhuman). These features are therefore perceived as more *distinctive* (e.g., Lawrence, 1949; Gibson, 1991; Goldstone, 1994; Campbell et al., 1997; for an overview of *acquired distinctiveness* and *acquired equivalence*, see e.g., Goldstone, 1998). In contrast to the warped profile on the human side of the DHL's category boundary, there was no such difference in PD for unambiguous within-category avatar faces compared with the ambiguous faces at or closest to the category boundary. Considered in terms of the CP literature, participants thus appear to be perceptually desensitized to information that would facilitate visual discrimination of within-category human faces, while a corresponding desensitization is not apparent within the nonhuman category.

The present study did not aim to show that PD within the nonhuman category can change with perceptual and categorization experience. But stimulus exposure and explicit categorization training is known to evoke changes in discrimination sensitivity to a range of stimuli, from simple line drawings of unnatural entities to perceptually complex facial stimuli (e.g., Gibson, 1991; Hall, 1991; Schyns and Murphy, 1994; Goldstone, 1996; Levin, 1996, 2000; Livingston et al., 1998; Stevenage, 1998; Goldstone et al., 2003; Kikutani et al., 2008, 2010). If categorization training can modulate PD performance along the DHL, this might induce effects of acquired equivalence, acquired equivalence, or both, resulting therefore in a different profile of warping along the DHL than shown in the present study. Presumably, categorization training would primarily influence the cognitive representation of the avatar side of the DHL. Training could be based, for example, on familiarization with avatar faces so that individuals learn to discriminate between these in terms of their unique features (Bruyer et al., 2004; McGugin et al., 2011). Alternatively, the impact of experience might be examined in designers. Animators, video game designers, and roboticists concerned about the uncanny effect and the impact of their designs on subjective affect (e.g., Minato et al., 2006; Walters et al., 2008; MacDorman et al., 2009) regularly expose themselves to a range of humanlike faces and actively engage in carefully crafting perceptual features related to human likeness. Differences between novices and experts in processing perceptual information has been reported for other domains of expertise, ranging from the diagnosis of aberrant structures in x-rays to identification of gender in chickens (e.g., Burns and Ward, 1978; Biederman and Shiffrar, 1987; Myles-Worsley et al., 1988; Peron and Allen, 1988; Norman et al., 1992). This has yet to be examined in the present context.

In view of this asymmetry in PD performance, the third experiment examined whether avatars are preferentially coded at the category level and human faces at the exemplar level. This idea draws on findings relating to the *other-race affect* that show greater accuracy recognizing individual own- compared with other-race faces and show less configural coding of out-group members (e.g., Rhodes et al., 1989, 2006). The third experiment thus used inverted faces because inversion strongly influences efficient configural coding of spatial relations (e.g., nose-mouth distance) among facial features (Leder and Bruce, 2000), while its impact on processing the individual features is generally much weaker (e.g., Murray et al., 2000). The lack of an inversion effect in the present experiment suggests that PD performance along the DHL generally relies more on coding human likeness-specifying information of facial features such as the eyes, nose, and mouth and other features such as skin tone rather than on coding the spatial relationship among these features, even though coding configural information might enhance the accuracy of coding facial features (Tanaka and Farah, 1993).

The potential role of these facial features in the reported asymmetry in PD is therefore worth considering in terms of the *avatar-feature hypothesis* (Cheetham et al., 2013). This hypothesis initially related to categorization performance along the DHL. It suggests that participants preferentially detect perceptual information in nonhuman faces that is diagnostic of the nonhuman category. Assuming that it is cognitively less demanding to detect the presence of this diagnostic information in avatars rather than its absence in human faces, a categorization decision strategy based on “avatar vs. not avatar” instead of “avatar vs. human” would result in faster categorization decisions for avatars (see also *feature asymmetry*, Treisman and Gormican, 1988). Consistent with this, the forced choice categorization data of all three experiments show shorter categorization response latencies for avatar compared with human faces, replicating the data of previous studies (Cheetham et al., 2013, 2011; see also Levin, 1996).

It is similarly possible that in the ABX tasks participants preferentially detected or found it easier to detect perceptual information that is diagnostic of human likeness specifically in the nonhuman faces of the DHL, thus facilitating the asymmetric effect in PD for these faces. The absence of an inversion effect in the ABX task indicates that this information is not likely to be relational (i.e., based on configural coding). Given that inversion effects are weaker for facial features like the eyes, nose and mouth and absent for facial properties like facial color (Leder and Carbon, 2006), it is conceivable that the participants coded and processed perceptual differences along the DHL on the basis of facial properties such as smoothed skin texture, color and shading. This does not exclude a role for feature-based processing, especially as processing for example the general luminance properties of faces can enhance processing of facial features (Sergent, 1986; Schyns and Oliva, 1994; Schyns and Gosselin, 2003). The question is why these properties should be easier to detect in the avatar faces. In view of the task context of processing novel avatars and everyday human faces, it is possible that perceptual information indicating the novelty of these facial properties renders this information more salient in the nonhuman faces of the DHL and that novelty therefore serves as a primitive perceptual feature that can facilitate PD within the avatar category (Levin, 2000). An alternative suggestion is that visual PD performance might be facilitated by the progressive reduction in perceptual complexity of the morphs with increasing distance from the human end of the continua independently of experience and perceptual strategy; the avatar parent faces have less human structural and textural detail than the human parent faces. Reduced humanlike complexity such as the reduced variance in shading of the smoothed skin texture might in itself provide a more easily detectable feature of these morphs that eases PD.

The UVH predicts that greater PD will evoke greater feelings of strangeness (i.e., feelings of less familiarity) at the point along the DHL at or near which ambiguity is greatest. The data of the second experiment suggest that this is wrong on two counts. Firstly, the analysis of familiarity ratings indicates that greater feelings of strangeness (i.e., feelings of less familiarity) are not reported for ambiguous faces. Instead, feelings of strangeness increased with increasing morph distance from the human end of the continua. This is consistent with the pattern reported in other studies in which comparably well-controlled morph continua and *ad hoc* measures of shinwakan such as measures of pleasantness have been used (e.g., Looser and Wheatley, 2010). These empirical data contradict the theoretical model of the UVH's uncanny valley effect presented by Moore (2012). Two drawbacks of that model is that it assumes *a priori* that the uncanny curve in Mori's graphical representation of the UVH is correct and it does not consider the potential impact of asymmetry in perceptual and categorization experience that is implicit in the UVH. The overall implication of the present familiarity data is that more humanlike stimuli simply evoke more positive affective experience and are preferred over less humanlike stimuli. The most straightforward explanation for this relates to the *mere-exposure* effect (Zajonc, 1968). This means that repeated exposure to human faces over a person's history of social interaction and the often more positive affective tone of interaction with particular in-groups results in more positive evaluations of other in-group members (e.g., Reis and Gable, 2003).

Second, the inter-individual differences approach adopted in the second experiment shows that there is indeed a significant relationship between familiarity and PD difficulty, but that the direction of this relationship is the opposite of that predicted in the UVH. Increasing PD difficulty is associated with more positive feelings of familiarity. The effect was evident for nonhuman and ambiguous faces, whereas there was no significant relationship between PD and feelings of familiarity for human faces. This correlative effect was greatest for ambiguous faces, indicating that, irrespective of the causal relationship between PD and feelings of familiarity, the UVH' prediction is most likely to be incorrect. It should be noted that the UVH does not suggest how DP difficulty and its affective dimension, shinwakan, should be operationalised. This issue has hampered uncanny-related research from the outset. But the approach taken in the present study to testing the relationship between DP and affective experience (as described in the UVH) was straightforward and produces strong effects, indicating that further examination of this relationship might be fruitful.

Why greater PD difficulty should correlate with more positive self assessment of affect is not clear. A popular account of the uncanny effect is based on the *Hedonic Fluency Model* (Winkielman et al., 2003; see Yamada et al., 2013). This suggests that negative evaluations of novel or unfamiliar stimuli relate to cognitive difficulty extracting information needed for rapid and efficient processing. This makes sense if the UVH' prediction for PD is assumed to be correct. But the present data suggest that this prediction is incorrect. The present PD data do however fit better with an alternative model of processing fluency, the *Fluency Amplification Model* (Albrecht and Carbon, 2014). This model states that processing fluency enhances the affective reaction that the stimulus already evokes. Assuming for example that the valence of a given stimulus is initially experienced as comparatively negative, individuals who experience greater fluency (in our case, lesser difficulty in PD) will experience the negative stimulus as even more negative. By the same token, greater PD difficulty would correlate with less negative ratings. While this interpretation is consistent with the present correlative data, further investigation of this finding and of the role of interindividual differences in state affect is needed.

The ABX PD task is useful for testing naive participants because it requires no description of the specific physical dimensions along which the stimuli vary and participants do not need to know the category labels. One explanation for CP effects suggests a role for the presence of category labels (Roberson and Davidoff, 2000; Pilling et al., 2003; Kikutani et al., 2008). This is because within-category stimuli differ only at the exemplar level, while cross-category stimuli differ both at the exemplar and category levels. If exemplar-level information and category-level information are processed in parallel so that the category boundary can be represented in naive participants after initial learning (Marsolek, 2004), category-level processing might encourage the use of labeling and the emergence of CP effects. This effect might be even stronger in a task with a strong memory component such as the ABX task (i.e., the test stimulus must be compared with the stored representation of the target stimuli). Considering the asymmetry in discrimination performance around the category boundary, a labeling effect is unlikely, unless labeling affected the human side of the category boundary only. It has however been argued that any impact of category labeling would be reflected in specific *within-category discrimination asymmetries* (Hanley and Roberson, 2011). There are no such asymmetries within the human or nonhuman categories.

In summary, the data of the three experiments reject the implicit assumption underlying the UVH' key prediction. The data show lesser PD difficulty for categorically ambiguous faces and for unambiguous avatar faces and, notably, greater PD difficulty for unambiguous human faces. The data indicate that this asymmetry in PD difficulty cannot be attributed to differences between human and nonhuman faces in configural coding. It is likely that perceptual differences along the DHL are generally processed on the basis of human likeness-related manipulations of facial properties such as skin texture, color and shading. Ratings of familiarity show that faces associated with greatest category ambiguity do not show an uncanny-like effect. Negatively valenced ratings increased across the tested stimulus conditions with increasing distance from the human end of the continua. An interindividual differences approach revealed that greater PD difficulty is associated with more positively rather than negatively valenced experience. This challenges the key idea behind the UVH. This effect is strongest for ambiguous faces, suggesting that this effect is more consistent with the metaphor “happy valley” and, correspondingly, the fluency amplification effect. These findings for our static faces thus indicate that both the assumed distribution of PD difficulty along the DHL and the predicted relationship between PD difficulty and affective experience (as “conceptualized” in the UVH) are very likely wrong.

Clearly, it is not possible to confirm or refute the vaguely formulated non-scientific UVH in its current form. Our approach has been to augment the notions underlying the UVH with the necessary assumptions needed to render the essential features of the hypothesis testable. While we find no evidence in favor of these notions, our findings do not exclude the possibility that alternative experimental paradigms and other methodologies might show effects consistent with the underlying idea of the UVH. It should be noted that only male face stimuli were presented. The choice of stimuli for this study was not guided by the well-known depiction of Mori's hypothesis in Figure 1 because we sought to ensure that perceptual discriminative, categorization and familiarity judgments would not be confounded by factors other than the manipulation of human likeness (for a discussion of confounds, see Cheetham and Jancke, 2013). This study presented stimuli similar to those used in preceding studies (Cheetham et al., 2011, 2013), which, given the absence of comparable paradigms in the investigation of the DHL, has provided an effective means to developing insight and a basis for further uncanny-related study. But an important element of further study would be to examine whether these findings generalize to other static stimuli. Whether these findings might apply to dynamic nonhuman characters (e.g., Saygin et al., 2012; Burleigh et al., 2013; Urgen et al., 2013) and to such characters in human interaction (e.g., Cheetham et al., 2009) is open to further investigation.

### Conflict of interest statement

The authors declare that the research was conducted in the absence of any commercial or financial relationships that could be construed as a potential conflict of interest.
