# Supplementary material for: Perceptual discrimination difficulty and familiarity in the Uncanny Valley: more like a “Happy Valley”
Source: Front Psychol. 2014 Nov 19;5:1219. doi: 10.3389/fpsyg.2014.01219 (PMC4237038; doi:10.3389/fpsyg.2014.01219)
Supplement: Supplementary file 1 [file DataSheet1.PDF]

Supplemental information: Perceptual discrimination difficulty and familiarity in the Uncanny Valley: More like a Happy Valley.

Marcus Cheetham, Pascal Suter, Lutz Jancke

Experiment 1

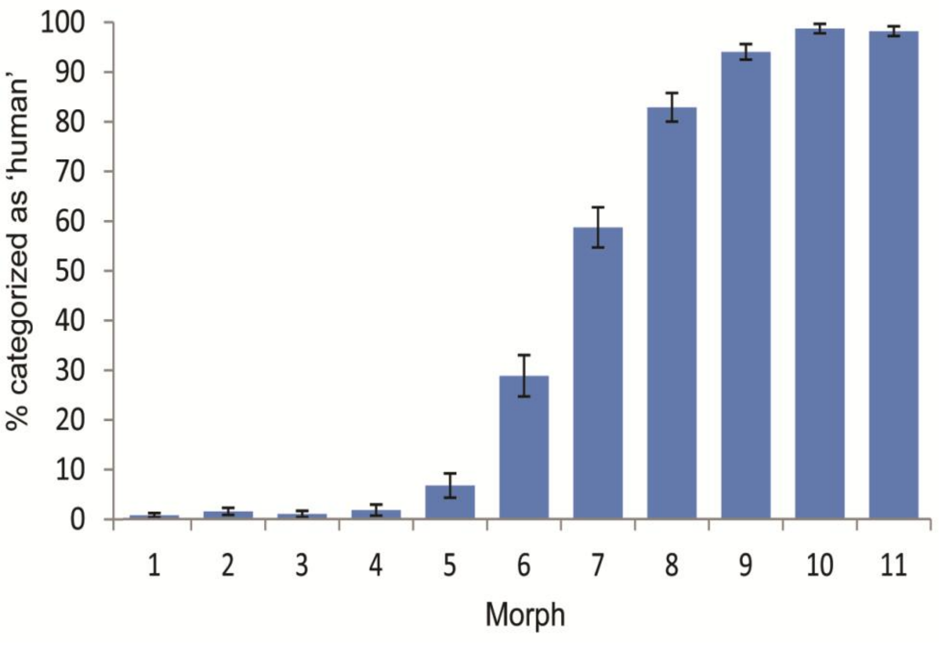

Supplemental Figure 1. Results of the forced choice classification task for responses. Mean responses are depicted in terms of % of 'human' responses for the different morph positions 1–11, with error bars indicating 1 SE (N = 49). The category boundary value was M = 6.95.

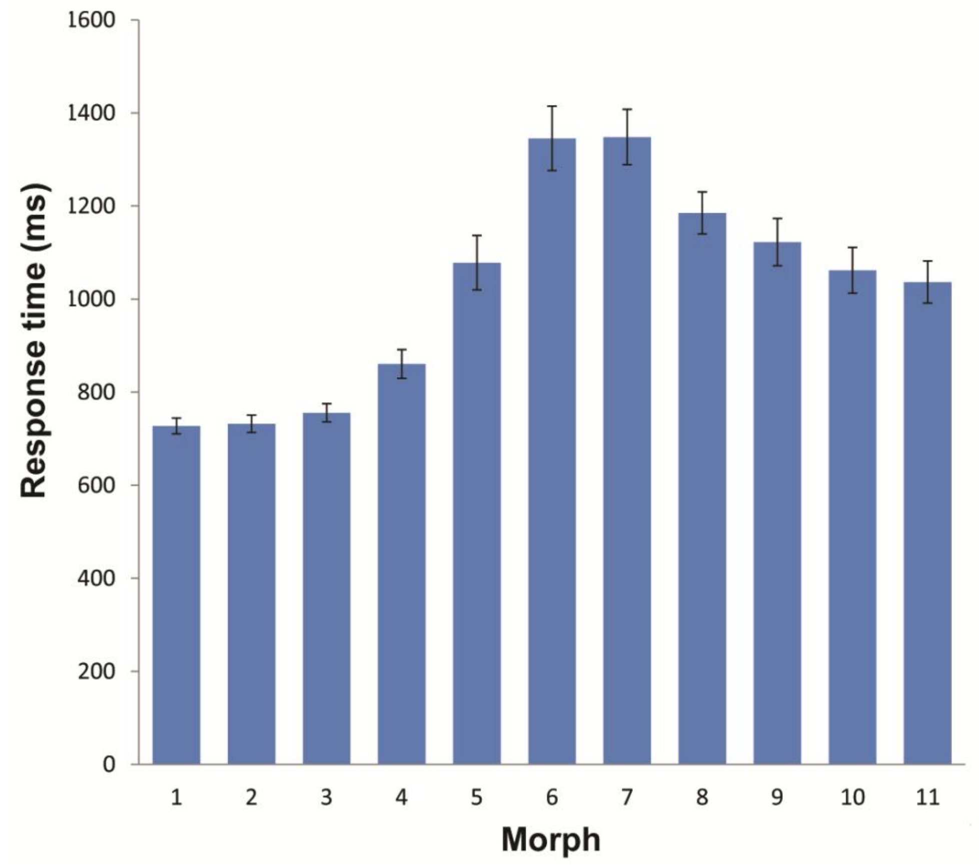

Supplemental Figure 2. Results of the forced choice classification task for RT. The mean aggregated RT data at the different morph positions 1–11, with error bars indicating 1 SE (*N* = 49), show enhanced RT for both the most ambiguous morph position M7 and its neighbor M6. The category boundary value was M = 6.95. There was no significant difference in RT between M6 and M7

Experiment 2

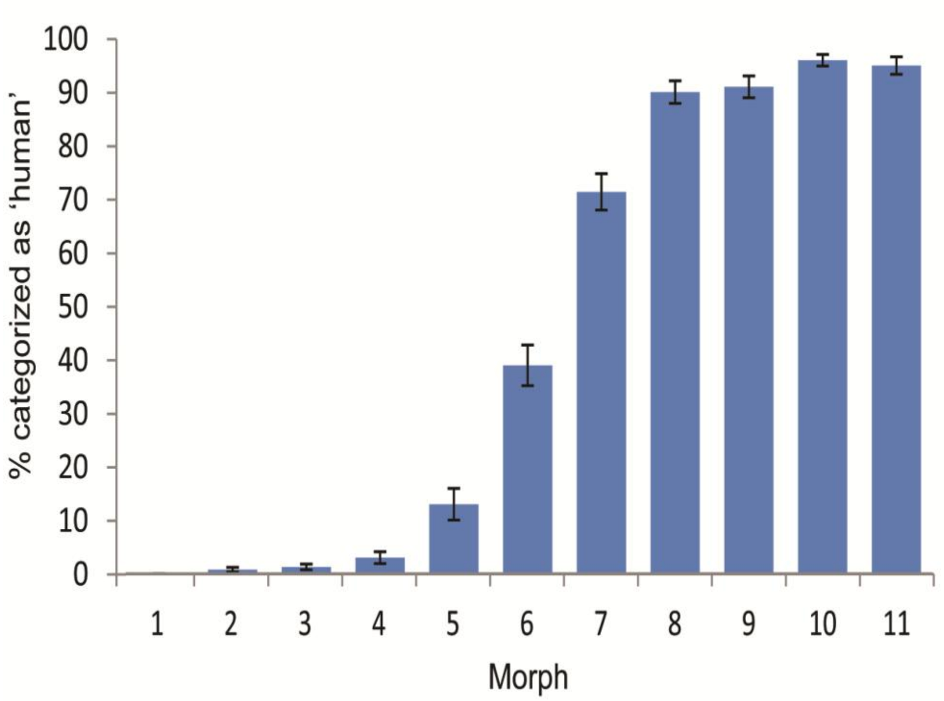

Supplemental Figure 3. Results of the forced choice classification task for responses. Mean responses are depicted in terms of % of 'human' responses for the different morph positions 1–11, with error bars indicating 1 SE (N = 49). The category boundary value was M = 6.6.

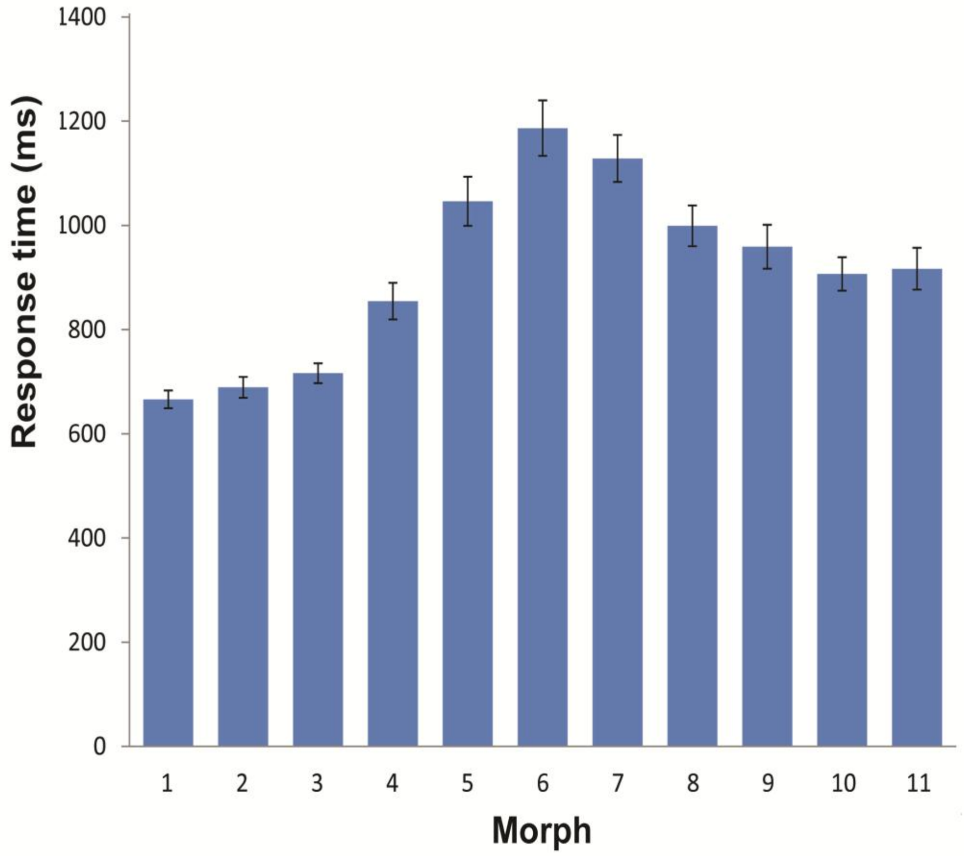

Supplemental Figure 4. Results of the forced choice classification task for RT. The mean aggregated RT data at the different morph positions 1–11, with error bars indicating 1 SE (*N* = 49), show enhanced RT for the most ambiguous morph position (M6). The category boundary value was M = 6.6.

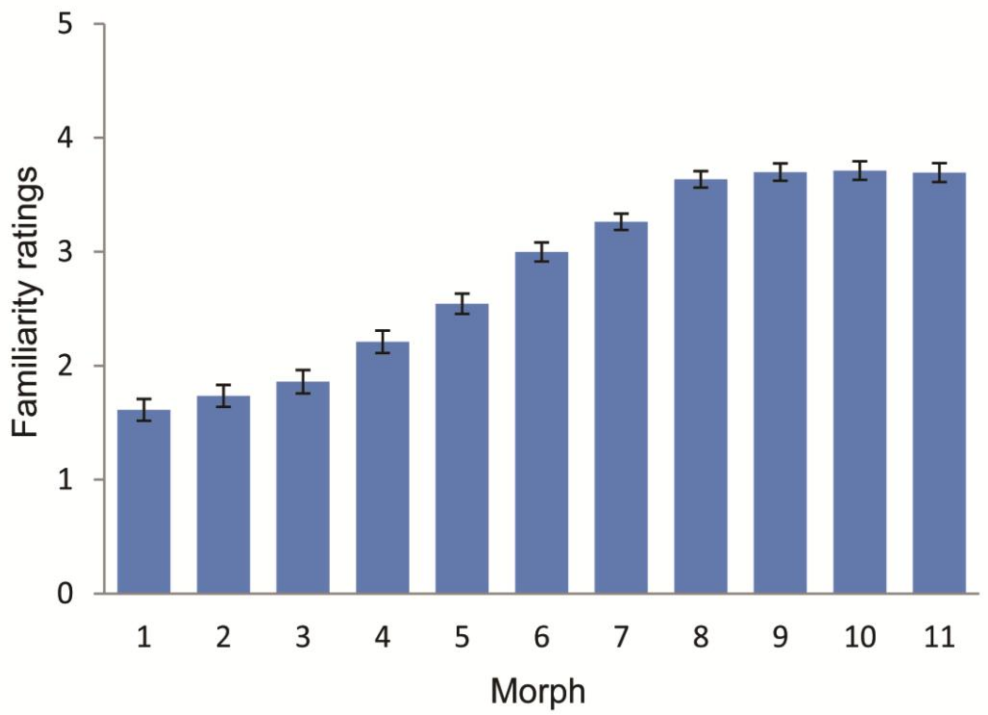

Supplemental Figure 5. Results of the familiarity ratings task. The mean rating data at the different morph positions 1–11, with error bars indicating 1 SE (*N* = 49), show enhanced feelings of familiarity with increasing human likeness. There is no indication of an uncanny effect.

Experiment 3

Categorisation responses

The slope of the categorisation response function was used to summarise the category judgements by fitting logistic function models to the data of each participant across continua. The parameter estimates derived from each model were entered in analyses of logistic function of categorisation responses and of the category boundary. For the logistic function, the parameter estimates were tested against zero in a one-sample t-test. The result shows a highly significant logistic component (t_{48}= 27.83, $p > 0.001$), this reflecting a logistic-shaped function consistent with the presence of a category boundary.

The parameter estimates derived from each logistic function model of each participant across continua were tested against zero in a one-sample t-test and showed a highly significant logistic component (t_{48}= 27.83, $p > 0.001$. Based on the parameter estimates β0 and β1, the mean category boundary value was $M = 6.6$ (see Supplemental Figure 5). Across continua, the most ambiguous face morph M6 is closest to this boundary.

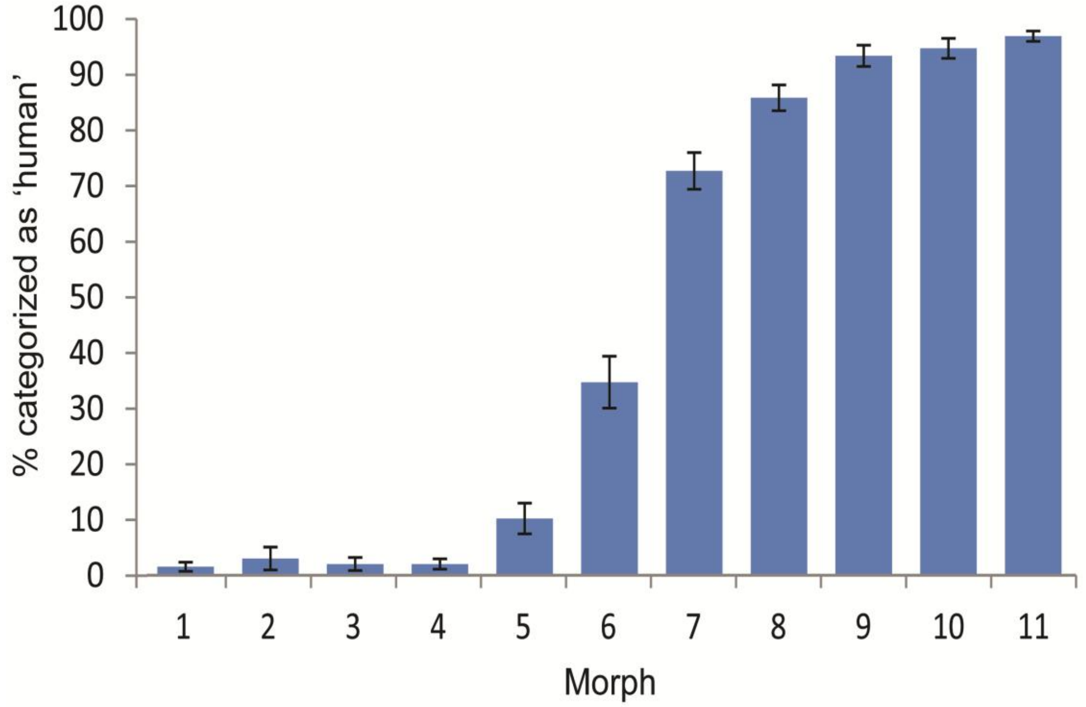

Supplemental Figure 6. Results of the forced choice classification task for responses. Mean responses are depicted in terms of % of 'human' responses for the different morph positions 1–11, with error bars indicating 1 SE (N = 25). The category boundary value was M = 6.7.

To compute the category boundary value (i.e., y=.5: -ln[β0]/ln[β1]), the estimates for the β0 and β1 parameters of each participant across continua were used. Using these parameters, the mean category boundary value was *M* = 6.6. The category boundary value indicates the point along the morph continua that corresponds with the ordinate midpoint between the lower and upper asymptotes, that is, the point of maximum uncertainty of 50% in categorisation judgements. Across continua, the face morph position M6 is closest to this boundary. informal inspection of the logistic-shaped curve in Supplemental Fig 3 shows a lower and upper asymptote of avatar and human categorisation responses nearing 100% in categorisation decision certainty for avatars and humans, respectively.

To show the effects of this profile of high and low ambiguity in categorization judgements more clearly, we tested for differences in category decisions between the unambiguous avatar (i.e., M2, M3, M4) and human faces (i.e., M8, M9, M10) and the most ambiguous faces (i.e., M6). Consistent with the approach in Experiment 1, the choice of morphs permitted control for physical morph distance along continua between M6 at the category boundary and the avatar and human faces.

A one-way *RM-ANOVA* was performed on the dependent variable mean 'categorization' response of each participant across continua, using the factor 'morph' position (3 levels: "M2, M3, M4", "M6", "M8, M9, M10"). This analysis showed a highly significant effect for morph position [$F(1.22, 58.52) = 483.72$, $p < 0.001$]. Categorisation difficulty for M6 was closest to chance level of 50% (*M* = 40.31; *SE*= 3.73), while that for the human faces was *M* = 93.58 (*SE* = 1.13) and for avatar faces *M* = 2.63 (*SE* =.44).

Categorisation response times

Differences in category ambiguity, as indicated by the logistic-shaped response function, are likely to be reflected in different *response times* (*RT*) for category judgements. Before data analysis, short RT

latencies of less than 100 ms were excluded. RT data for long latency outliers were screened by z-standardizing and filtering out data points using z=3 as a cut-off score. Analyses were conducted with and without outliers. These analyses produced the same pattern of results. The findings are therefore reported for the complete data set. Confirming RT differences in category decision difficulty, a one-way RM-ANOVA with morph position (11 levels: M1- M11) and RT as the dependent variable showed a main effect for morph position, $F(4.58, 220.22) = 39.03$, $p < 0.001$.

The longest response latencies would be expected to correspond with the morph position closest to the category boundary, that is, at M6 (see Supplemental Fig 6). A one-way RM-ANOVA analysis with 'morph' positions (3 levels: M2-M4, M6, M8-M10) and RT in ms as dependent variable was conducted. The analysis showed a highly significant effect for morph position, $F(2, 96) = 54.99$, $p < 0.001$. Pre-planned contrasts showed that RT was longer significantly longer for human ($M = 957$, $SE = 34$) than for avatar faces ($M = 751$, $SE = 23$), $F(1,48) = 15.72$, $p > 0.001$. Pre-planned contrasts showed also that RT for M6 ($M = 1191$, $SD = 53$) differed highly significantly from RT for the other morph positions ($M = 851$, $SD = 0.37$), $F(1,48) = 65.91$, $p < 0.001$.

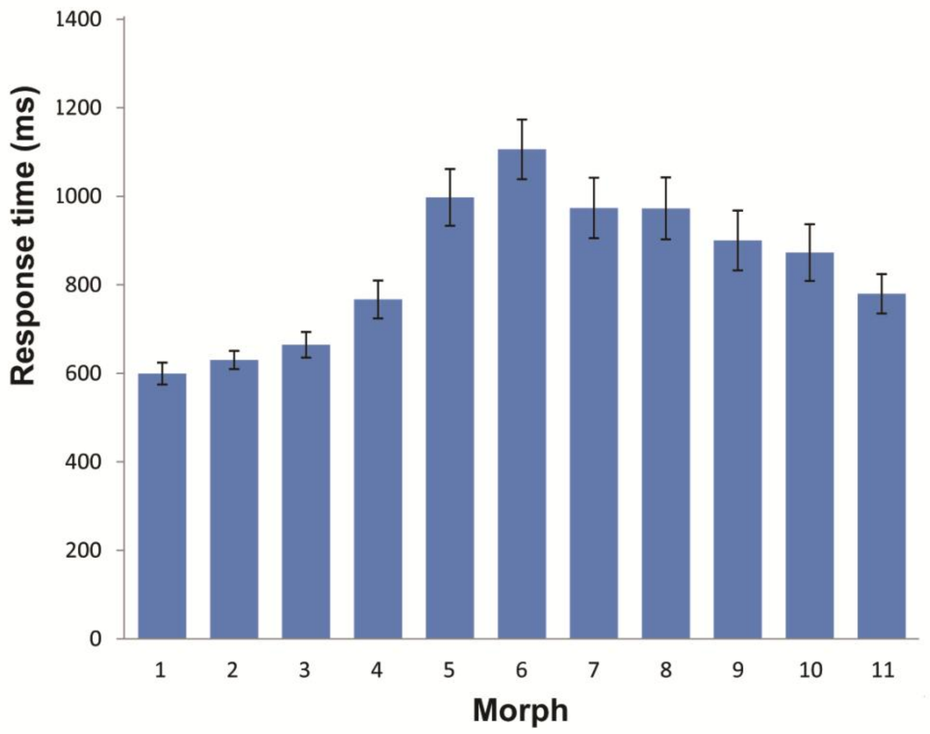

Supplemental Figure 7. Results of the forced choice classification task for RT. The mean aggregated RT data (blue line) at the different morph positions 1–11, with error bars indicating 1 SE ($N = 25$), show enhanced RT for the most ambiguous position (M6).

Experiments 1, 2 and 3

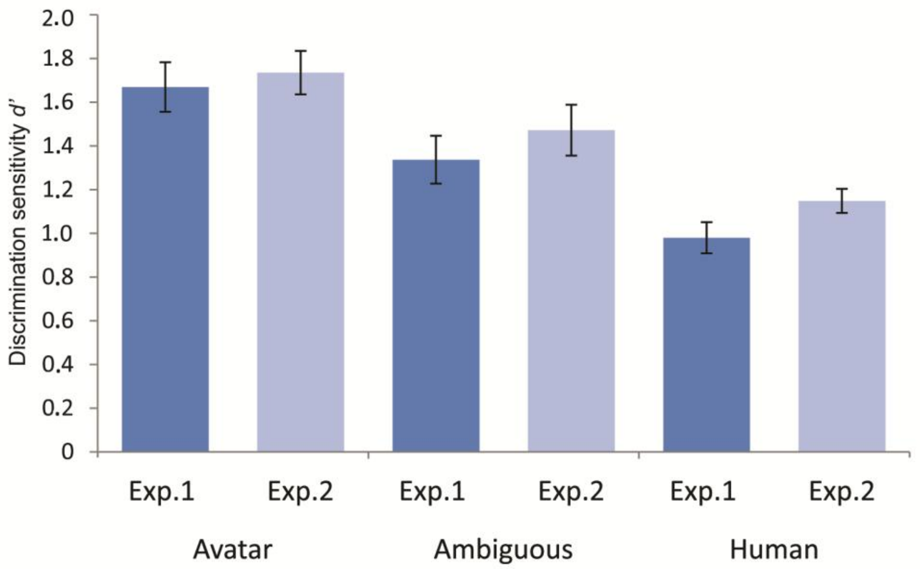

Supplemental Figure 8. Results of the ABX perceptual discrimination task of Experiments 1 and 2. This figure illustrates mean discrimination sensitivity d' in the ABX perceptual discrimination task for the avatar, ambiguous and human faces. Error bars indicating 1 SE for Experiment 1 (*N* = 49) and Experiment 2 (*N* = 49)

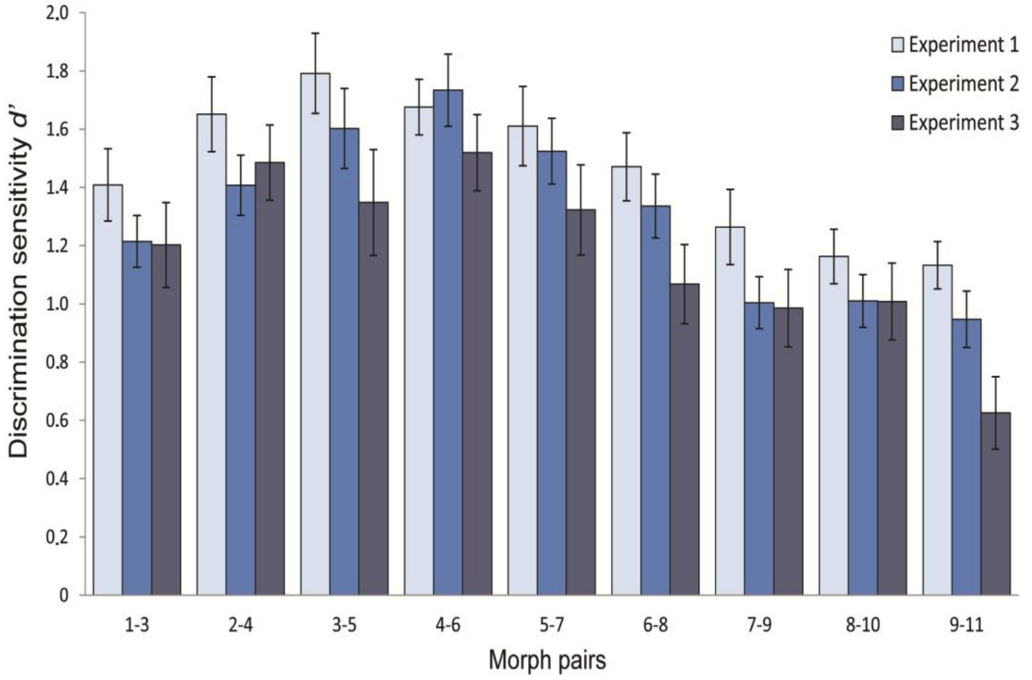

Supplemental Figure 9. Results of the ABX perceptual discrimination task of all ABX Experiments. This figure illustrates mean discrimination sensitivity d' in the ABX perceptual discrimination task for each of the morph pairs along the continua. Error bars indicating 1 SE for Experiment 1 (*N* = 49), Experiment 2 (*N* = 49), and Experiment 3 (*N* = 25).
